# *Lycium barbarum* Polysaccharides: Extraction, Structural Characteristics, Anti-Inflammatory Mechanisms, Safety Profiles and Applications

**DOI:** 10.3390/ijms27167213

**Published:** 2026-08-13

**Authors:** Jiaming Bai, Jiani Fu, Yuanyuan Huang, Quan Liu, Jingya Mo, Yuanxiang Zhang, Bei Zhou, Yanchun Wu, Jingquan Yuan

**Affiliations:** Institute of Chinese Medicine and Zhuang-Yao Ethnic Medicine, Guangxi University of Chinese Medicine, Nanning 530200, China

**Keywords:** *Lycium barbarum* polysaccharides, extraction technology, structural characterization, anti-inflammatory mechanism, toxicological safety, pharmaceutical and healthcare application

## Abstract

*Lycium barbarum* polysaccharides (LBPs), the primary bioactive components extracted from the traditional medicinal and edible plant *Lycium barbarum* L., have attracted extensive attention in biomedical research due to their superior anti-inflammatory, immunomodulatory and biological safety properties. Inflammation is a key pathological basis of various chronic metabolic and immune diseases, and LBPs can exert targeted regulatory effects on inflammatory responses through multiple molecular pathways. This article systematically reviews the latest advances in extraction technologies and structural characterization of LBPs, and illustrates their regulatory mechanisms against inflammation in different organs. Relevant toxicological studies are also summarized to confirm their low toxicity and safety. Additionally, it comprehensively concludes the current progress of LBPs in healthcare products, pharmaceutical development, and related patent innovations. Future perspectives highlight green efficient extraction, structural modification, targeted delivery, and in-depth mechanism exploration, aiming to provide a comprehensive theoretical basis for the further development and industrial application of LBPs.

## 1. Introduction

**Figure 1:**
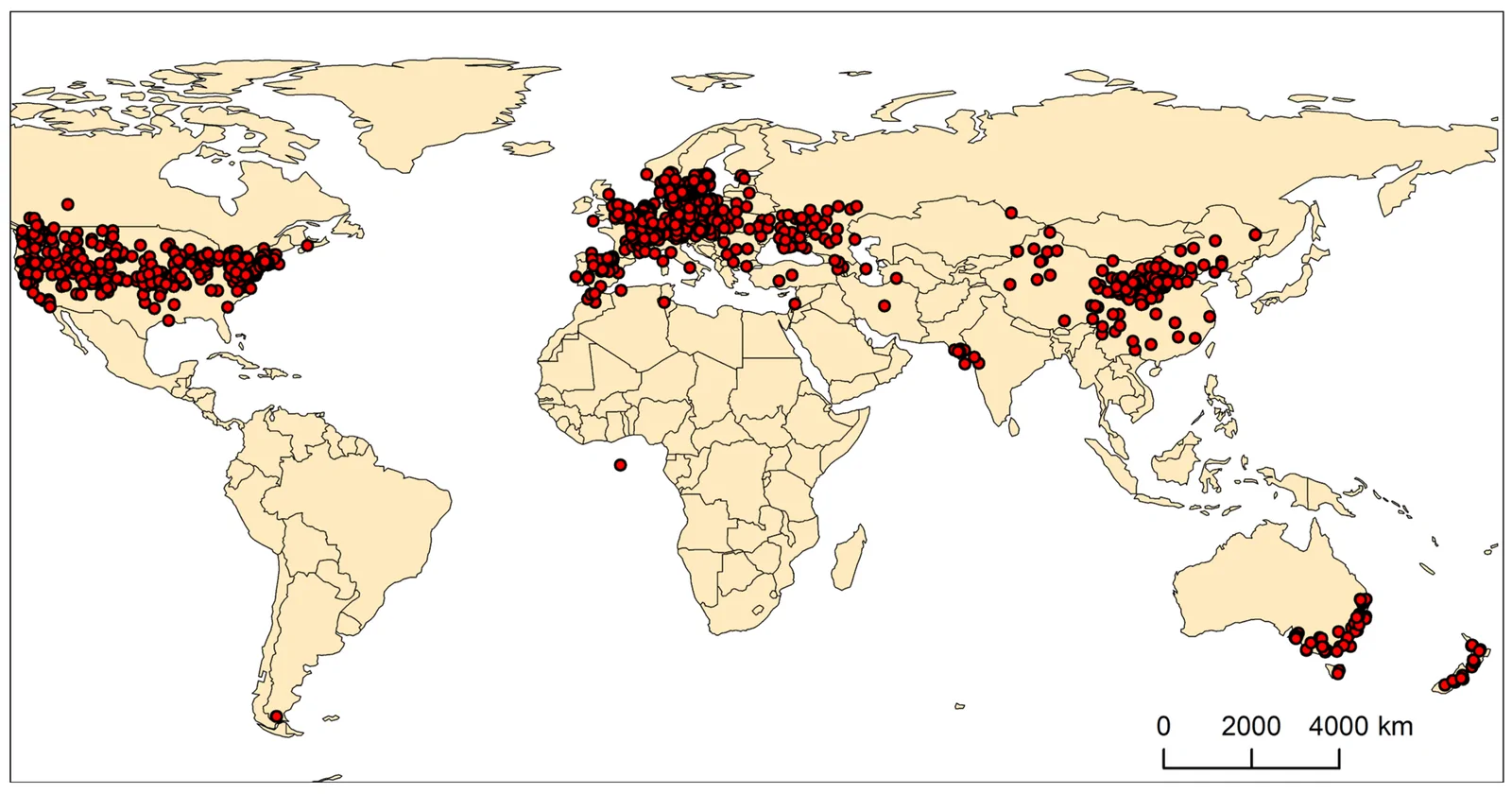
Global distribution of *L. barbarum.* (This distribution map is independently redrawn by the authors using ArcGIS software (version 10.8.2) based on public longitude and latitude occurrence data downloaded from GBIF [4]. The original GBIF dataset was released under the CC BY-NC 4.0 license, and this recreated artwork belongs to the authors with full copyright for publication.).

**Figure 2:**
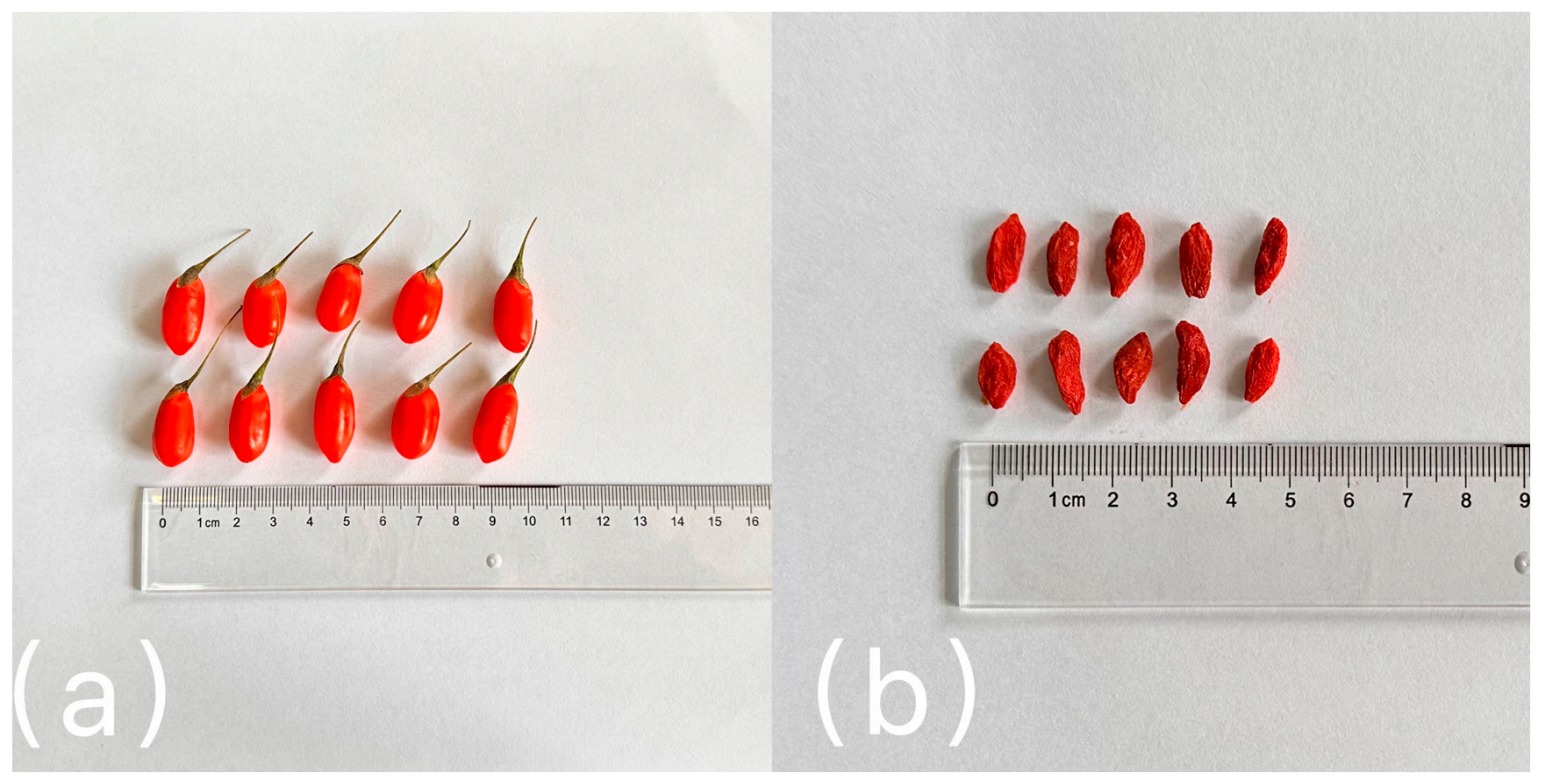
(**a**): Images of fresh L. barbarum. (**b**): Images of dried L. barbarum. Panels (**a**,**b**) are original illustrations drawn independently by the authors of this study.

*Lycium barbarum* L., a perennial herb belonging to the genus *Lycium* of the Solanaceae family, has several common names, including “Xi Goji”, “Shan Goji”, and “Bai Ci” [1]. Globally, *L. barbarum* has been distributed across multiple countries in Europe, Asia, Africa, South America, and North America [2]. Native to northern and central China, *L. barbarum* is mainly distributed in northwestern provinces, including Xinjiang Uygur Autonomous Region, Inner Mongolia Autonomous Region, Tibet Autonomous Region, Qinghai Province, Gansu Province, and Ningxia Hui Autonomous Region. Among these regions, Ningxia Hui Autonomous Region is recognized as the global origin of *L. barbarum* [3]. The global distribution of *L. barbarum* (a species of the *Lycium* genus) was visualized via ArcGIS relying on geographic coordinate data from GBIF [4], as displayed in Figure 1. In the *L. barbarum* plant, the *L. barbarum* fruit (Gou qi zi) refers to its dried ripe fruit, which is oval-shaped, orange-red, approximately 2 cm in length, and has a sweet taste. *L. barbarum* prefers to grow in well-drained weakly alkaline soil and under sufficient sunlight [5]. The fresh and dried fruits of *L. barbarum* are shown in Figure 2.

In the ‘Food Hygiene Law of the People’s Republic of China (Trial Implementation)’ issued in 1982, Goji berry (*L. barbarum* fruit) was officially recognized and included in the first batch of “traditional Chinese medicines that are both food and medicine”. In ‘Shen Nong Ben Cao Jing (Shennong’s Classic of the Materia Medica, Han Dynasty, c. 206 BCE–220 CE)’, the earliest extant pharmaceutical monograph in China, Goji berry was classified as a “top-grade” (shang pin) medicinal herb. It is stated therein that “long-term consumption of Goji berry leads to a light body, resistance to aging, and tolerance to cold and heat” [6]. This description not only elaborates on the medicinal effects of Goji berry but also highlights the health benefits of its long-term consumption, laying a historical foundation for its subsequent use as a substance with the dual property of “food-medicine homology” in traditional Chinese medicine. Furthermore, ‘Materia Medica for Dietary Therapy’ records that Goji berry “strengthens the tendons, resists aging, dispels pathogenic wind, nourishes the muscles and bones, benefits the human body, and relieves consumptive disease” [7]. This record explains the health-preserving and healthcare functions of Goji berry from the perspective of dietary therapy, further embodying its dual characteristic of being usable both as food and medicine. In China, Goji berry has long been widely used as an edible fruit in traditional Chinese medicine (TCM), with its therapeutic effects extensively applied in traditional Chinese herbal practice. It has also been included in China’s traditional pharmacopeias [8]. According to pharmacopeia records: Goji berry refers to the dried ripe fruit of *L. barbarum* L. The fruit is harvested in summer and autumn when it turns red, then processed through hot-air drying followed by removal of fruit stalks; alternatively, it is air-dried until the peel wrinkles, then sun-dried and stripped of fruit stalks. In TCM terminology, Goji berry is characterized by “sweet taste” (wei gan) and “neutral nature” (xing ping), and it “enters the Liver Meridian and Kidney Meridian” (gui Gan Jing, Shen Jing). Its pharmacological effects include “nourishing the liver and kidney, replenishing essence, and improving eyesight” (zi bu gan shen, yi jing ming mu). Clinically, it is indicated for the treatment of “consumptive disease with essence deficiency, soreness and pain of the waist and knees, dizziness and tinnitus, impotence and spermatorrhea, internal heat with thirst, blood deficiency with sallow complexion, and blurred vision” [9,10,11,12].

Goji berry is nutrient-rich and abundant in natural products, such as *Lycium barbarum* polysaccharides (LBPs), betaine, flavonoids, carotenoids, and vitamins. As a typical “food-medicine homology” substance in traditional Chinese medicine, it serves both dietary and medicinal purposes [13]. In recent years, due to its nutritional properties, goji berry has gained increasing popularity in the Western world; it is even promoted as a “superfood” in Europe and North America, making it one of the most extensively studied species within the *Lycium* genus [14]. Among its bioactive components, LBPs have attracted growing attention owing to their diverse biological activities and broad application prospects. LBPs refer to a class of natural products with multiple bioactive properties extracted from goji berry fruits [15]. Recognized as the most critical bioactive component of goji berry, LBPs account for 5–8% of the total dry matter in the fruits [16].

In recent years, with the in-depth research on LBPs, their roles in anti-inflammation, immunomodulation, antioxidant activity, anti-tumor effects, and blood glucose regulation have been gradually elucidated [17,18,19,20]. However, there is currently a lack of systematic and comprehensive reviews focusing on its anti-inflammatory effects. This article summarizes the structural characteristics and extraction methods of LBPs, elaborates on the anti-inflammatory effects of LBPs on inflammation in different organs (including the eyes, kidneys, bones and joints, liver, lungs, and colon), reviews relevant toxicity studies, and sorts out the research progress of LBPs from two application dimensions: the food and healthcare field and the biomedical field. For the food and healthcare field, this review discusses the potential of LBPs as natural functional ingredients to improve inflammatory sub-health and prevent chronic inflammatory disorders [21]. For the biomedical field, we illustrate the prospect of LBPs as anti-inflammatory adjuvants to alleviate multi-organ inflammatory damage via multiple signaling pathways [22]. The purpose of this review is to provide new insights for the development of novel anti-inflammatory drugs and offer references for further exploration of the biological activities of LBPs.

**Figure 3:**
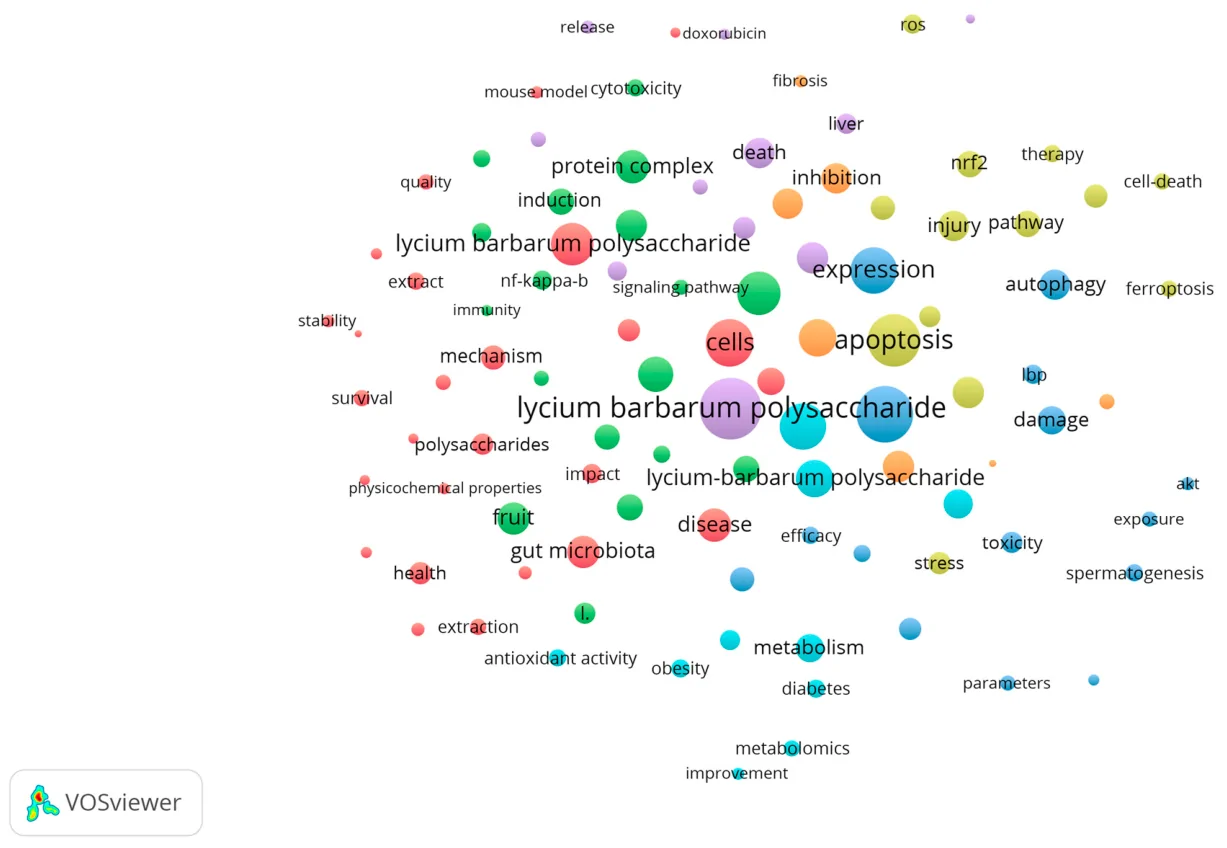
*L. barbarum* polysaccharides keyword co-occurrence network diagram.

To objectively and comprehensively illustrate the global research status, developmental context and cutting-edge hotspots in the field of LBPs, electronic databases Web of Science, PubMed and CNKI were retrieved to gather published literature relevant to this review. The major English search terms included *Lycium barbarum* polysaccharides, extraction, purification, anti-inflammatory mechanism, toxicology, healthcare applications and patents. Corresponding Chinese keywords were used for literature retrieval in Chinese databases. Review articles and original research papers were included, whereas conference abstracts and non-academic popular articles were excluded. Quantitative analysis was performed via VOS viewer from multiple dimensions such as publication output, journal distribution, highly cited references and keyword co-occurrence networks, so as to sort out research priorities and emerging trends and provide scientific evidence for subsequent basic research and translational application of LBPs. The keyword co-occurrence network of LBPs obtained from bibliometric analysis is presented in Figure 3.

## 2. Structural Characteristics of LBPs

In terms of structure, the monosaccharide composition of LBPs mainly includes arabinose, mannose, rhamnose, glucuronic acid, galacturonic acid, glucose, and galactose. Among these, glucose, galactose, and arabinose are the main monosaccharide units forming the backbone of LBPs, and the monosaccharides are linked by glycosidic bonds [23]. As a highly branched polysaccharide, the structural backbone of LBPs is mainly composed of (1→4)-*β*-Galp, (1→3)-*β*-Galp, (1→6)-*β*-Galp, (1→6)-*α*-glucan, and (1→4)-*α*-polygalacturonic acid ester. It has various branched and terminal structures, with a molecular weight ranging from 8 to 214 kDa [24]. The anti-inflammatory performance of LBPs is highly dependent on their intrinsic structural features. Monosaccharide composition is a pivotal factor governing bioactivity; a high proportion of arabinose and galactose significantly strengthens the inhibitory effects of LBPs on inflammatory cascades, whereas appropriate galacturonic acid content is critical for ameliorating intestinal inflammation. LBPs fractions possessing a backbone of *β*-1,3/1,4-galactoside linkages and *α*-1,5-arabinose side chains display superior anti-inflammatory potency at a branching degree of 0.3–0.6, a structural conformation that favors immune receptor recognition and binding. Moreover, LBPs with a uniform molecular weight of 20–80 kDa and a compact spherical conformation present better water solubility and elevated bioavailability. Furthermore, moderate phosphorylation and sulfation modifications can effectively improve the anti-inflammatory potential of LBPs.

The structure of LBPs exhibits high diversity. Their molecular structure characterization mainly includes relative molecular weight, monosaccharide composition, functional groups, types and linkage modes of glycosidic bonds, and spatial structure. In addition, fruit polysaccharides extracted from *Lycium ruthenicum* Murr. (a plant belonging to the genus *Lycium*, the same genus as *L. barbarum* L.), namely *L. ruthenicum* Murr. Polysaccharides (LRPs), have attracted increasing attention in the research exploring their molecular structural features. This is because LRPs share commonalities with LBPs in terms of monosaccharide composition, main chain structural modules, and spatial conformation. Table 1 summarizes key structural parameters of purified LBPs and LRPs that have been reported in existing studies, covering molecular weight, monosaccharide composition, quantity ratio of substance, and structures (backbone).

## 3. Extraction Methods for LBPs

**Figure 4:**
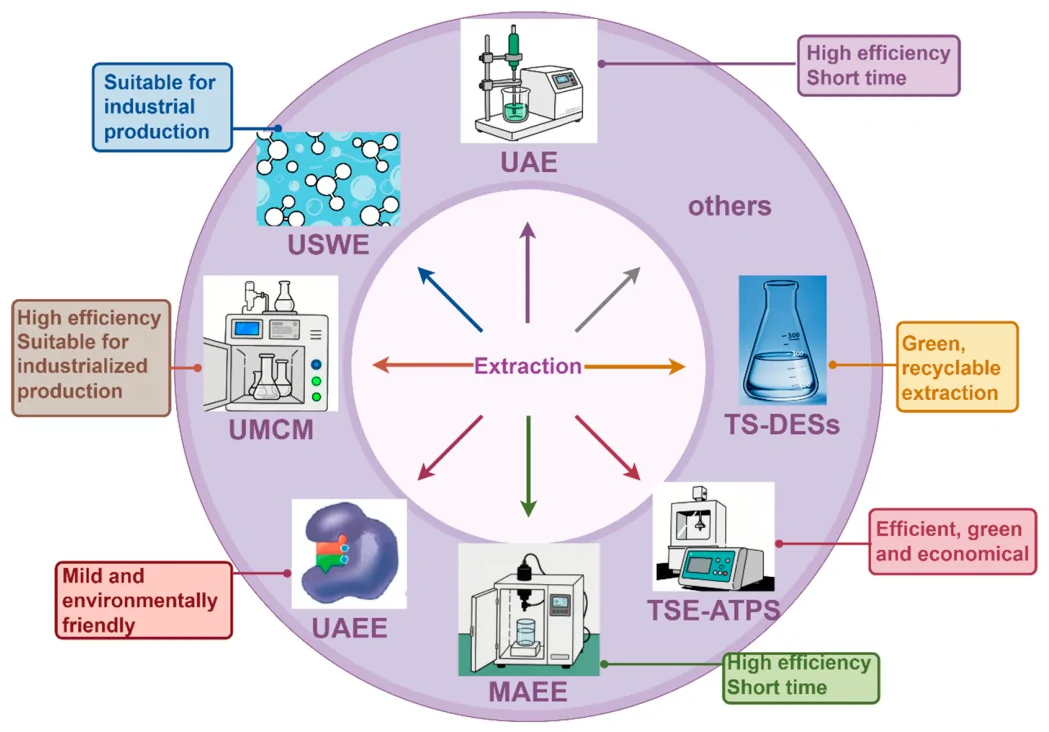
Optimized Extraction Process of LBPs.

Polysaccharides from medicinal plants typically exist as complex mixtures. Extraction is not only the first step but also a crucial step in the research of medicinal plant polysaccharides [45]. Currently, several common methods are used to extract polysaccharides from goji berries: Hot Water Extraction (HWE), which is simple to operate and has a low extraction cost [46]; Enzymatic-Assisted Extraction (EAE), which features mild extraction conditions, high efficiency, and better preservation of the original biological activity of the polysaccharides [47]; Ultrasound-Assisted Extraction (UAE), which offers high extraction efficiency, short extraction time, low solvent consumption, and enhanced biological activity of the polysaccharides [48]; Microwave-Assisted Extraction (MAE), which is time-saving, energy-efficient, and environmentally friendly, provides uniform heating, and yields a high recovery rate of active ingredients [49]; and Ethanol Extraction, which is particularly effective in preserving the original biological activity of the polysaccharides [50]. However, these methods also have limitations: HWE is time-consuming, inefficient, and energy-intensive [51]. EAE requires stringent operating conditions and high production costs. UAE demands specialized equipment and is not suitable for large-scale production [52]. MAE is not suitable for thermally sensitive materials. Therefore, with technological advancements, many extraction methods have been developed to optimize conditions and improve yield and selectivity. The optimized extraction methods of LBPs and their corresponding characteristics are shown in Figure 4.

### 3.1. Ultrasound Assisted Extraction (UAE)

#### 3.1.1. Response Surface Methodology (RSM)-Optimized UAE

UAE exploits the cavitation effect of ultrasound to disrupt plant cell walls, thereby enhancing mass transfer and increasing the extraction yield of target compounds. This method is notable for its short extraction time, low operating temperature, and high efficiency [53]. Song et al. [54] optimized the UAE method using response surface methodology (RSM). By combining single-factor experiments with RSM, they determined the optimal extraction conditions for goji berry polysaccharides as follows: an extraction time of 80 min, an extraction temperature of 73 °C, a solid-to-liquid ratio of 1 g:38 mL, and an ultrasonic power of 185 W. Under these conditions, the optimal extraction yield of goji berry polysaccharides was 12.54 ± 0.12%. RSM-optimized UAE improves the extraction yield of LBPs. The extraction duration is only 47% of that required by conventional HWE, and the optimized extraction temperature is lower, which effectively avoids high-temperature-induced degradation.

LBPs are thermosensitive bioactive compounds. Prolonged exposure to high temperatures breaks the glycosidic bonds of polysaccharides, degrades macromolecular polysaccharide chains, and disrupts their advanced spatial conformation. Long-duration hot-water maceration also facilitates side reactions including oxidation and hydrolysis of polysaccharides. The mild low-temperature and short-duration operating conditions of RSM-optimized UAE efficiently preserve the intact molecular structure of thermolabile LBPs. Multiple biological functions of LBPs, such as immunomodulation, anti-tumor effects, and hypoglycemic activity, are strongly dependent on specific molecular weight distributions and intact branched-chain structures; UAE maintains these physiological activities well. Meanwhile, this technique shortens the thermal residence time of polysaccharides in aqueous solution and alleviates oxidative damage, thereby retaining the antioxidant and anti-aging capacities of LBPs to the maximum extent. Nevertheless, UAE exhibits a narrow applicable processing window. Excessively high ultrasonic power or prolonged extraction time triggers intense cavitation and strong shear forces, which fracture long polysaccharide chains of LBPs and destroy their spatial conformation. Additionally, harsh ultrasonic conditions cause massive dissolution of impurities that bind to LBPs, ultimately decreasing polysaccharide purity and biological activity [55]. Accordingly, strict regulation of optimal process parameters is indispensable throughout the extraction procedure.

#### 3.1.2. Ultrasound-Assisted Subcritical Water Extraction (USWE)

Zhao et al. [56] utilized ultrasound-assisted subcritical water extraction (USWE) to extract LBPs. The extraction conditions were as follows: temperature, 100 °C; time, 53 min; solid-to-liquid ratio, 26 mL/g; pressure, 5 MPa; and ultrasonic power, 160 W. Under these conditions, the yield of LBPs was 5.728%. Subcritical water, defined as the liquid phase of hot water maintained under sufficient pressure, possesses temperature-dependent selectivity, environmental friendliness, high efficiency, and low cost [57]. USWE integrates the dual merits of high-temperature/high-pressure subcritical water treatment and ultrasonic cavitation. This green and efficient technique employs only water as the extraction solvent and eliminates the prerequisite of pulverizing *L. barbarum* raw materials. Compared with conventional HWE, single UAE, and independent subcritical water extraction (SWE), USWE delivers markedly higher recovery yields of LBPs with shortened extraction duration, rendering them more suitable for continuous industrial manufacturing.

The synergistic combination of ultrasound and subcritical water exclusively disrupts plant cell walls without inducing excessive depolymerization of polysaccharide macromolecules. Furthermore, the coupled extraction system facilitates the co-elution of low-molecular-weight antioxidant LBPs fragments, alongside phenolic compounds and flavonoids that exert synergistic antioxidative effects. Such co-extracted bioactive co-components substantially reinforce superoxide anion radical scavenging capacity, enabling full retention or even enhancement of the antioxidant potential of LBPs. Additionally, the USWE protocol preserves the reductive functional groups of polysaccharide backbones; these hydroxyl moieties are essential for DPPH radical scavenging performance, and their structural integrity is well maintained throughout the extraction workflow. Nevertheless, the USWE system requires a hermetically sealed reactor to sustain high-temperature and high-pressure operating conditions. Excessively elevated pressure compresses raw feedstock, which impedes sufficient contact between the extraction solvent, ultrasonic field and plant matrix. The simultaneous modulation of multiple interdependent process parameters poses substantial technical challenges. Moreover, USWE relies on specialized integrated equipment equipped with pressurization, temperature control and ultrasonic modules, which features intricate mechanical architecture and incurs higher capital investment costs.

#### 3.1.3. Ultrasound-Microwave Extraction (UMCE)

Microwave irradiation induces a heating effect that accelerates the dissolution of endogenous plant cells. When combined with ultrasound, it enhances the accessibility of polysaccharides and improves extraction yield [58]. Na et al. [59] utilized a combined ultrasound-microwave extraction (UMCE) method to extract polysaccharides from goji berry leaves. The optimal conditions were as follows: microwave time of 16 min, ultrasound time of 20 min, particle size of 100 mesh, and liquid-to-solid ratio of 55:1. Under these conditions, the extraction yield of goji berry leaf polysaccharides (LLP) reached 1.873%. Dual synergistic cell disruption and heat transfer can be realized via the coupling of ultrasonic cavitation and microwave volumetric heating, which markedly elevates the recovery yield of polysaccharides. Benefiting from the superimposed synergistic effects of the two physical external fields, this hybrid extraction eliminates the requirement for prolonged static maceration and drastically shortens the overall extraction cycle. Water is exclusively adopted as the extraction medium, rendering the process cost-effective, environmentally benign, free of organic solvent residues, and contamination-free.

The dual ultrasonic–microwave cell lysis facilitates the full solubilization of polysaccharide fractions with diverse molecular weights. Low-molecular-weight polysaccharide fractions are enriched with bioactive functional moieties and exhibit potent free radical scavenging capacity. Moreover, the coupled extraction process avoids excessive polysaccharide depolymerization and preserves critical active sites including hydroxyl and aldehyde groups on polysaccharide backbones, as well as the intact primary polysaccharide chain structures. Such structural integrity enables effective inhibitory effects against starch digestive enzymes and retards carbohydrate hydrolysis, conferring prominent in vitro hypoglycemic potential upon the obtained polysaccharides. Nevertheless, UMCE is governed by multiple nonlinear interdependent variables, and its implementation relies on specialized integrated composite extraction equipment. These characteristics impose stringent demands on hardware configuration and operational proficiency, restricting its practical deployment primarily to industrial-scale continuous production rather than small-scale laboratory batch extraction.

### 3.2. Enzymatic Assisted Extraction (EAE)

#### 3.2.1. Ultrasound-Assisted Enzymatic Extraction (UAEE)

Studies have shown that the use of cellulase or pectinase during the extraction of LBPs can promote cell wall degradation, thereby facilitating the release of polysaccharides [60]. Moreover, the combination of cellulase and protease can enzymatically hydrolyze proteins that are bound to polysaccharides, releasing more polysaccharides and thereby increasing the yield of polysaccharides [61]. Liu et al. [62] optimized the ultrasound-assisted enzymatic extraction (UAEE) of LBPs using RSM. The optimal extraction conditions were as follows: extraction time, 20.29 min; ultrasound output power, 78.6 W; cellulase concentration, 2.15%; and extraction temperature, 55.79 °C. Under these conditions, the actual yield of polysaccharides was 6.31 ± 0.03%. UAEE operates via two synergistic mechanisms, achieving drastically enhanced extraction efficiency. Its extraction duration accounts for merely 22% of that required by single enzymatic extraction, which substantially cuts time consumption and thermal energy input, balancing high processing efficiency and favorable recovery yield of LBPs.

Cellulase specifically degrades cellulose components in plant cell walls without cleaving the glycosidic bonds of polysaccharides. Consequently, the intact macromolecular skeleton and functional hydroxyl moieties of LBPs are well preserved, whereby the intrinsic pharmacological activities of LBPs—including immunomodulatory, antioxidant, and hypoglycemic capacities—remain unimpaired throughout extraction. Nevertheless, cellulase exhibits poor thermal stability and tends to undergo rapid inactivation under harsh extraction conditions. Excess cellulase dosage also introduces extraneous impurities into crude extracts. Furthermore, the commercial cost of cellulase significantly raises raw material expenses during large-scale industrial manufacturing.

#### 3.2.2. Microwave-Assisted Composite Enzymatic Method (MAEE)

Chen et al. [63] utilized a microwave-assisted composite enzymatic method (MAEE) to extract LBPs. The enzyme mixture comprised cellulase, pectinase, and protease in a mass ratio of 3:1:2, with an enzyme loading of 7% based on the mass of goji berries. The enzymatic hydrolysis was conducted for 2.50 h at 52 °C and pH 5.2, followed by microwave treatment for 4 min. Under these conditions, the yield of LBPs was 24.37% ± 1.65%. MAEE relies on synergistic triple enzymatic hydrolysis coupled with rapid microwave thermal perturbation. Cellulase degrades the fibrous skeletal network of plant cell walls, pectinase hydrolyzes intercellular pectin substances, and protease dissociates protein moieties bound to polysaccharide chains. The multi-mechanism synergistic effect simultaneously releases intracellular polysaccharides, remarkably elevating the recovery yield of LBPs and shortening overall extraction duration.

Within the MAEE workflow, the compound enzyme cocktail exclusively targets structural components of cell walls, and short-duration microwave irradiation does not disrupt the macromolecular backbone of polysaccharides. Antioxidative functional groups such as hydroxyl residues are fully retained, alongside diversified bioactive polysaccharide fractions co-eluted during extraction. Consequently, LBPs isolated via MAEE exhibit superior scavenging capacities against DPPH and ABTS cation radicals relative to extracts obtained from conventional extraction protocols, manifesting markedly reinforced antioxidant potency. Nevertheless, the triple-enzyme system imposes stringent restrictions on multiple processing parameters, including microwave exposure duration, enzymatic hydrolysis temperature, and medium pH, with a narrow feasible window for parameter matching. In addition, the high commercial cost of compound enzyme preparations and complex operational procedures collectively limit the scalability of MAEE for large-scale industrial manufacturing.

### 3.3. Other Extraction Methods

Hu et al. [64] developed an efficient method for extracting LBPs using tissue disruption extraction (TDE) in combination with an aqueous two-phase system (ATPS). TDE is an innovative technique that rapidly fragments target particles while vigorously stirring the solution. This method offers several advantages, including high extraction yield, low solvent consumption, low energy requirements, simple operation, and extremely short extraction time [65]. ATPS are generally composed of polymers and salts or short-chain alcohols and salts. These systems support environmental protection, continuous operation, and scalability. Their low viscosity and high mass transfer efficiency make them widely applicable [66]. The optimal extraction process was determined by combining single-factor experiments with response surface methodology. The conditions included an aqueous two-phase system (ATPS) composed of 17.86% (NH_4_)_2_SO_4_ and 28.86% ethanol, a solid-to-liquid ratio of 1:30 (*m*/*v*), tissue disruption at 8000 rpm, and an extraction time of 4 min. Under these conditions, the yield of LBPs was 2.479%. TSE-ATPS integrates the dual strengths of high-speed shear-mediated cell disruption under ambient temperature and synchronous separation via aqueous two-phase systems. The entire extraction procedure is completed within merely 4 min without high-temperature treatment. This technique yields remarkably higher recovery of LBPs compared with Hot Water Extraction and conventional ultrasound-assisted extraction. In addition, most impurities can be separated in a single-step operation, which greatly streamlines subsequent purification workflows. The extraction media are environmentally benign, rendering this approach readily scalable for industrial applications.

Deep eutectic solvents (DESs), initially proposed by Abbott, are composed of hydrogen bond donors (HBDs) and hydrogen bond acceptors (HBAs) [67]. In recent years, DESs have emerged as a green solvent alternative to organic solvents and ionic liquids and have been widely applied in the extraction and separation of various bioactive compounds [68]. Tang et al. [69] developed temperature-switchable deep eutectic solvents (TS-DESs) for the extraction of LBPs. Using a 70 wt% DES-3 (tetracaine: lauric acid, 1:1) solvent, a solid-to-liquid ratio of 1:25 g/mL, and an extraction temperature of 35 °C, the extraction was performed for 70 min, achieving a maximum LBPs extraction yield of 46.5%. TS-DESs represent a class of recyclable and eco-friendly extraction media. Efficient solubilization of LBPs is achieved at temperatures below the critical solution temperature (CST). When the system temperature exceeds the CST, facile phase separation occurs to facilitate solvent recovery, which drastically cuts overall solvent consumption. Furthermore, prominent LBPs extraction yields can be attained under the optimized processing conditions of TS-DES-based extraction.

TSE-ATPS and TS-DESs enable low-temperature extraction, which fully conserves anti-inflammatory key acidic sugar moieties such as galacturonic acid and prevents thermally induced cleavage of polysaccharide glycosidic bonds. In addition, phase separation effects of the two systems selectively enrich low-molecular-weight polysaccharide fractions, thereby comprehensively enhancing the anti-inflammatory bioactivity of recovered LBPs. Nevertheless, both extraction techniques require precise control over multiple process parameters and involve elaborate, complicated operational workflows accompanied by prolonged total extraction durations. These drawbacks collectively raise capital and operational costs when scaled up to industrial mass production.

With the continuous innovation and upgrading of modern extraction and separation technologies, the traditional extraction limitations of LBPs, including low extraction yield, high energy consumption and poor purity, have been effectively improved. Current LBPs extraction research mainly focuses on process optimization and green and efficient transformation. On the one hand, RSM is widely adopted in relevant research to optimize pivotal processing parameters including extraction temperature, extraction duration, and liquid–solid ratio. Such statistical optimization remarkably elevates the recovery yield of LBPs and improves technological stability. Meanwhile, excessive depolymerization of polysaccharide macromolecules can be avoided, and their intact molecular architectures are well preserved, whereby core pharmacological activities such as immunomodulation and anti-inflammatory potency remain unimpaired throughout extraction. On the other hand, hybrid-assisted extraction techniques represented by UMCE and UAEE exert synergistic effects integrating physical field-mediated cell disruption and biochemical enzymatic hydrolysis of plant cell wall matrices. This dual synergistic mechanism efficiently ruptures plant cellular structures and accelerates the solubilization of intracellular polysaccharides. Moreover, the intact structural features of recovered LBPs endow the extracts with potent free radical scavenging capacity, further boosting overall extraction efficiency and selectivity toward target polysaccharide fractions. In addition, the green solvent replacement method replaces traditional toxic and inefficient extraction solvents, conforming to the concept of green and sustainable biomanufacturing. In summary, modern optimized extraction technologies achieve effective balance and coordination among high yield, high efficiency, low energy consumption and environmental friendliness, providing reliable technical support for the large-scale preparation and industrial application of high-purity LBPs. The process parameters of these extraction methods are presented in Table 2.

## 4. Anti-Inflammatory Effects of LBPs on Different Organs

Inflammation is a response to cell and tissue damage caused by pathogens, physical injury, or chemical exposure [70]. It is an immune stress response. It serves as a protective mechanism of the body against external stimuli, infections, endogenous tissue damage, and other insults, and occurs extensively in cells, tissues, and organs throughout the body [71]. While acute inflammation is a protective immune response, chronic or excessive inflammation can lead to various inflammatory diseases [72]. Therefore, alleviating and reducing inflammatory responses in the body is beneficial for promoting overall health. *L. barbarum* is rich in a variety of bioactive components, among which LBPs have demonstrated significant anti-inflammatory activity in recent studies. The following elaborate on the anti-inflammatory effects of LBPs on inflammation in different organs (including the eyes, kidneys, bones and joints, liver, lungs, and colon) as well as their underlying mechanisms. As shown in Table 3.

### 4.1. Anti-Inflammatory Effect of LBPs on Eyes

#### 4.1.1. Chemical Corneal Injury

Liu et al. [73] investigated the repair and protective effects of LBPs on corneal injury induced by NaOH alkali burns. To establish an alkali-burn corneal injury model, SD rats were used. Local anesthesia was induced by instilling hydrochloric lidocaine onto the corneal surface. A filter paper soaked in 1 M NaOH solution was then applied to the central cornea of the right eye for 30 s, followed by rinsing the burn site and conjunctival sac with normal saline for 1 min. To investigate the therapeutic effects of LBPs on corneal injury, a saline solution containing LBPs at a concentration of 2 mg/mL was administered as local eye drops to the rats. The study demonstrated that LBPs exert protective effects against corneal injury in rats via upstream suppression of proinflammatory cytokine secretion (*p* < 0.01). Mechanistically, LBPs significantly downregulate the mRNA transcription of JUN, CASP3 and MMP9 (*p* < 0.01), thereby blocking downstream inflammatory cascades, cellular apoptosis and stromal fibrotic responses triggered by the IL-17 signaling axis.

Qin et al. [74] investigated the therapeutic effects of LBPs on dry eye disease (DED) in mice. To induce DED, C57BL/6 mice were subjected to desiccating stress (DS) in combination with topical application of 0.3% benzalkonium chloride (BAC). To investigate the therapeutic effects of LBPs on corneal injury, the rats received daily topical administration of 5 µL LBPs eye drops at concentrations of 0.625, 2.5, or 12.5 mg/mL. The study found that LBPs at a concentration of 12.5 mg/mL exhibited the most effective alleviation of corneal inflammation in DED. LBPs significantly reduce the relative mRNA expression level of TNF-*α* in corneal tissues (*p* < 0.05). Meanwhile, LBPs block the phosphorylation and degradation of I*κ*B as well as the phosphorylation of MAPK family proteins, thereby curtailing the production of pro-inflammatory mediators including IL-6, IL-10 and MMPs. Such molecular cascades ultimately alleviate inflammatory cell infiltration into corneal tissue with remarkable statistical significance (*p* < 0.0001) and mitigate inflammatory damage in dry eye corneas.

#### 4.1.2. Inflammatory Injury of Human Keratinocytes

Li et al. [75] investigated the protective effects of LBPs against UVB-induced photodamage in human keratinocytes (HaCa T cells). Immortalized HaCa T cells were subjected to UVB irradiation using a UVB-TL/12 lamp at a dose of 300 mJ/cm^2^ to establish a photodamage cell model. LBPs were administered to the cells by adding it to the culture medium at a concentration of 300 µg/mL. The study demonstrated that LBPs protect HaCa T cells from UVB-induced photodamage by inhibiting the increased phosphorylation of p38 MAPK, which in turn reduces the activation of caspase-3 and the expression of MMP9. Consequently, LBPs suppress the production of inflammatory cytokines and attenuate the propagation of inflammation-related signaling pathways, thereby alleviating UVB-induced inflammatory responses.

#### 4.1.3. Ischemic Corneal Injury

In recent years, nanomedicine—an emerging interdisciplinary field that combines nanotechnology with pharmaceutical and biomedical sciences—has introduced new characteristics to drugs, including enhanced bioavailability and pharmacokinetics, reduced toxicity and adverse effects, controlled release, and minimized dosing requirements [76]. Consequently, Ni et al. [77] conducted a study in which they introduced the hydrophobic pinacol ester of phenylboronic acid (PBA)—a moiety with reactive oxygen species (ROS)-scavenging capacity—into the LBP molecule. This modification led to the formation of LBPs-derived nanoparticles (PLBP), which were designed to investigate their protective effects on visual function in a mouse model of retinal ischemia–reperfusion (IR) injury. The IR model was established in C57BL/6 mice by inserting a 30-gauge needle, connected to a standard saline reservoir positioned at a height of 1.2 m, into the anterior chamber of the left eye for 1 h, while avoiding damage to the corneal endothelium, iris, and lens. The drug was administered via intravitreal injection of 2 µL of PLBP solution at a concentration of 10 mg/mL into the left eye of the mice. The study demonstrated that PLBPs suppress the activity of IKK kinases and reduce IKK phosphorylation. Meanwhile, PLBPs restrain the ubiquitination and degradation of I*κ*B-*α* protein, which further blocks the nuclear translocation of p65 subunit. This cascade ultimately downregulates the transcriptional expression of pro-inflammatory cytokines including IL-6 and TNF-*α*, alleviates inflammatory responses induced by IR, and preserves visual function in IR model mice.

### 4.2. Anti-Inflammatory Effects of LBPs on the Kidneys

#### 4.2.1. Diabetic Nephropathy (DN)

Diabetic nephropathy (DN) is a prevalent complication of diabetes mellitus and the leading cause of chronic renal failure and end-stage renal disease. The pathogenesis of DN is extremely intricate, with persistent inflammatory response and renal fibrosis serving as the pivotal pathogenic contributors. Accordingly, anti-inflammatory mechanisms play a vital role in renal protection against DN [72]. Wan et al. [78] examined the renal protective effects of LBPs in a DN model induced by a high-fat diet combined with streptozotocin (HFD/STZ). Their findings further substantiate the critical role of LBP’s anti-inflammatory properties in mitigating DN. The DN mouse model was induced by feeding C57BL/6 mice a high-fat diet, followed by intraperitoneal injection of 0.1 mL/100 g body weight of a 0.1 M citrate buffer (pH 4.5) containing 40 mg/kg STZ. LBPs were administered via oral gavage at doses of 40 mg/kg, 80 mg/kg, and 160 mg/kg. The study demonstrated that LBPs at a dosage of 80 mg/kg provided optimal protection against renal inflammatory injury. LBPs downregulate both mRNA and protein levels of TNF-*α* in renal cortex tissue, thereby diminishing the initial stimulatory signals that trigger NF-*κ*B pathway activation. Meanwhile, LBPs restore the protein abundance of I*κ*B*α* and block the nuclear translocation of NF-*κ*B p65 subunit, which lowers the accumulation of activated intranuclear p65. Such molecular suppression further inhibits the transcription of downstream pro-inflammatory cytokines represented by IL-6, alleviates local inflammatory infiltration in renal tissue, and ultimately ameliorates inflammatory injury in DN kidneys.

#### 4.2.2. Chemically Induced Nephritis

Huang et al. [79] examined the effects of LBPs on renal inflammatory injury induced by lipopolysaccharide (LPS) in a rat model of sepsis. Sepsis was induced in Sprague–Dawley (SD) rats via intraperitoneal injection of LPS at a dose of 5 mg/kg. LBPs were administered via oral gavage at three different doses: 200 mg/kg, 400 mg/kg, and 800 mg/kg. Xie et al. [80] examined the therapeutic efficacy of LBPs in lead-induced renal injury in mice. The renal injury model was established in ICR mice by oral gavage with a lead acetate solution at a dose of 25 mg/kg. LBPs were administered via oral gavage at three doses: 200 mg/kg (low), 400 mg/kg (medium), and 600 mg/kg (high). The results of both experiments demonstrated that LBPs attenuate the Keap1-mediated degradation of Nrf2, which elevates the total intracellular abundance of Nrf2 protein. Accumulated Nrf2 reciprocally represses NF-κB inflammatory signaling cascades and curtails the secretion of multiple downstream pro-inflammatory cytokines. This reciprocal crosstalk between Nrf2 and NF-κB ultimately mitigates renal inflammatory infiltration and alleviates renal function impairment. In the study by Huang et al., the protective effects of LBPs against septic renal injury were dose-dependent, with the most pronounced protection observed at a dose of 800 mg/kg. In the study by Xie et al., the middle dose of 400 mg/kg LBPs showed significant efficacy in attenuating renal injury and improving renal function.

### 4.3. Anti-Inflammatory Effects of LBPs on Joints

Chronic inflammatory arthritis is characterized by the infiltration of macrophages and lymphocytes into the synovium, resulting in increased local production of pro-inflammatory cytokines, chemokines, and enzymes. This process further exacerbates the inflammatory synovial environment and activates potentially destructive signaling pathways, thereby compromising joint integrity [81]. Consequently, anti-inflammatory therapy is a critical strategy in the management of chronic inflammatory arthritis. Liu et al. [82] examined the therapeutic effects of LBPs on collagen-induced arthritis (CIA) in DBA/1 mice. The CIA model was established by subcutaneously injecting 200 µL of an emulsion containing bovine type II collagen (C-II) mixed with an equal volume of complete Freund’s adjuvant (CFA) at the base of the tail. LBPs were administered via intraperitoneal injection at three doses: 25 mg/kg (low), 50 mg/kg (medium), and 100 mg/kg (high). Lai et al. [83] examined the effects of LBPs on type II collagen-induced rheumatoid arthritis (RA) in Wistar rats. The RA model was established by injecting 0.2 mL of an emulsion containing an equal volume of C-II and incomplete Freund’s adjuvant (IFA) at a site 1.5 cm from the tip of the rat’s tail. LBPs were administered via oral gavage at a dose of 400 mg/kg.

Both studies demonstrated that LBPs downregulate the expression of upstream pro-inflammatory cytokines TNF-*α* and IL-6, which further curtails the secretion of chemokine MIP-1*α*. Such sequential molecular inhibition markedly suppresses the levels of bone-degrading MMPs, alleviates local inflammatory infiltration in joint tissues, and preserves the structural integrity of bone tissue, thereby attenuating inflammatory responses in CIA and RA. In addition, several studies have shown that within the gut microbiota, the abundance of specific beneficial bacteria is negatively correlated with the levels of pro-inflammatory cytokines and positively correlated with those of anti-inflammatory cytokines [84]. LBPs remodel the composition of intestinal microbiota: they reduce the relative abundance of pathogenic bacteria while elevating the proportion of beneficial commensal microbes. This gut microbial modulation restricts the generation of systemic inflammatory mediators and relieves systemic inflammatory status, ultimately ameliorating clinical symptoms of CII-triggered CIA and RA. In the study by Liu et al., the high dose of LBPs (100 mg/kg) was found to be more effective in alleviating the inflammatory response associated with arthritis.

**Figure 5:**
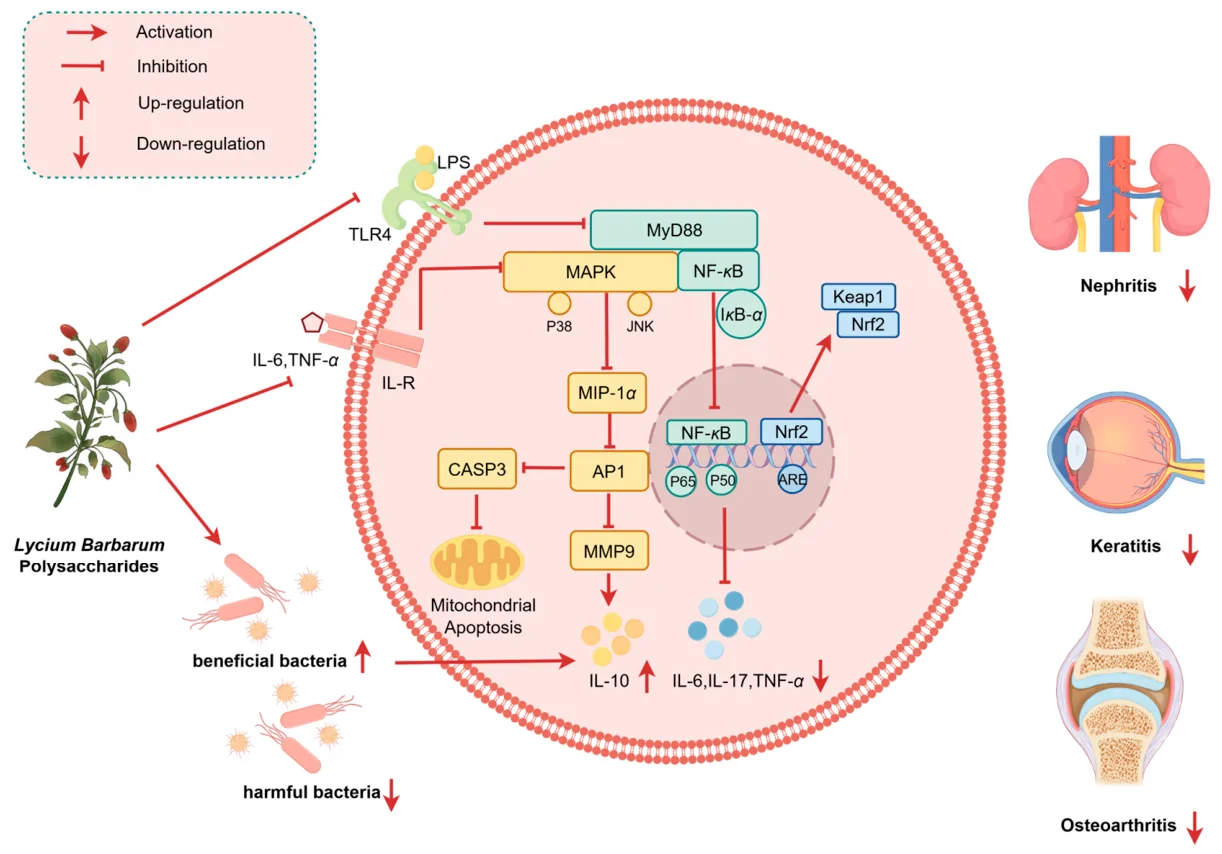
The main anti-inflammatory mechanism of LBPs in the eye, kidney and bone joints.

Collectively, LBPs exert prominent protective anti-inflammatory effects on eyes, kidneys, and bone joints, via multi-target and multi-pathway regulatory mechanisms. At the molecular level, LBPs significantly downregulate the mRNA expression of pro-inflammatory and apoptosis-related genes such as CASP3, TNF-*α*, and MMP9, while suppressing the overexpression of pivotal pro-inflammatory mediators, including IL-6, IL-17 and TNF-*α*. In terms of signaling regulation, LBPs efficiently block the excessive activation of the NF-*κ*B and MAPK signaling cascades, thereby mitigating inflammatory infiltration and tissue damage. Moreover, LBPs can reshape the intestinal microbial community structure by reducing the relative abundance of harmful bacteria and enriching beneficial flora, which further alleviates systemic inflammatory responses through the gut–organ axis. In general, the synergistic regulation of gene expression, core inflammatory signaling pathways, and intestinal microecology constitutes the essential molecular basis for the multi-organ protective and anti-inflammatory capacities of LBPs. The main anti-inflammatory mechanisms of LBPs in the eye, kidney, and bone joint are illustrated in Figure 5.

### 4.4. Anti-Inflammatory Effects of LBPs on the Liver

#### 4.4.1. Non-Alcoholic Steatohepatitis (NASH)

Non-alcoholic steatohepatitis (NASH) develops from the progression of simple steatosis in non-alcoholic fatty liver disease (NAFLD). As the most prevalent chronic liver disease, it affects approximately 25% of the global population. In advanced cases, NASH can further progress to liver cirrhosis and hepatocellular carcinoma, posing severe threats to human health [85]. Xiao et al. [86] examined the effects of LBPs on liver injury associated with methionine and choline-deficient (MCD) diet-induced non-alcoholic steatohepatitis (NASH). NASH was induced in C57BL/6 mice by feeding them an MCD diet for 6 weeks. LBPs were administered via oral gavage as a PBS solution containing 1 mg/kg LBPs. The study demonstrated that LBPs suppress IKK-mediated degradation of I*κ*B*α*, which blocks the nuclear translocation of NF-*κ*B p50 subunit and diminishes the transcriptional activity of NF-*κ*B. Meanwhile, LBPs hinder the assembly of NLRP3 inflammasome and impede the maturation and secretion of IL-1*β* and IL-18. Coordinately, such dual regulatory effects restrain the amplification of hepatic inflammatory cascades, ameliorate inflammatory responses, and alleviate liver injury in NASH.

Aerobic exercise (AE) is currently recognized as a primary intervention for NASH. Research indicates that AE training can reduce intrahepatic triglyceride levels and visceral fat content in patients with NAFLD, thereby exerting a protective effect on the liver [87]. Therefore, both studies by Li et al. [88] and Gao et al. [89] utilized LBPs in combination with AE to explore their effects on NASH induced by a high-fat diet. The NASH model was established in Sprague–Dawley rats by feeding them a diet containing 60 kcal% fat. The interventions included oral gavage of 100 mg/kg LBPs combined with AE training at a speed of 20 m/min for 60 min/d, and oral gavage of 50 mg/kg LBPs combined with AE training at a speed of 33 cm/s for 60 min/d. The results demonstrated that the combination of LBP+AE impedes the binding interaction between lipopolysaccharide (LPS) and TLR4, and downregulates the protein expression of TLR4 and MyD88. This regulatory effect further reduces the phosphorylation and nuclear translocation of NF-*κ*B p65 subunit, thereby interrupting intracellular inflammatory cascade signaling. Consequently, the secretion of pro-inflammatory cytokines including IL-6, IL-1*β* and TNF-*α* is markedly suppressed, which ultimately alleviates pathological manifestations of NASH.

#### 4.4.2. Chemically Induced Hepatitis

Xiao et al. [90] examined the protective effects of LBPs on CCl_4_-induced necrotic inflammation in the livers of C57BL/6N mice. Acute hepatotoxicity was induced by intraperitoneal injection of 50 µL/kg CCl_4_, with assessments conducted after 8 h. LBPs were administered orally at doses of 1 mg/kg and 10 mg/kg. The study demonstrated that pretreatment with 1 mg/kg LBPs significantly attenuated CCl_4_-induced hepatic necrosis. This attenuation was achieved through LBPs directly suppressing the DNA-binding activity of NF-*κ*B p50/p65 heterodimers and block their nuclear translocation and transcriptional functions. This inhibitory action significantly downregulates both mRNA and protein levels of downstream pro-inflammatory cytokines TNF-*α* and IL-1*β*, thereby interrupting inflammatory cascades and mitigating hepatic inflammatory lesions.

Cai et al. [91] examined the protective effects of LBPs on EtOH-induced hepatocyte injury. Normal human liver cells (L-02 cells) were exposed to 100 μg/mL of EtOH for 24 h to induce liver injury. The cells were then treated with LBPs at concentrations of 12 µg/mL, 24 µg/mL, and 48 µg/mL for 24 h. The results demonstrated that LBPs at a concentration of 48 µg/mL provided the most significant protection against hepatocyte injury (*p* < 0.01). This protective effect is derived from the fact that LBPs downregulate the protein expression of NLRP3, caspase-1 and GSDMD-N and inhibit NLRP3 inflammasome activation. This process reduces the release of proinflammatory cytokines IL-1*β* and IL-18, alleviates inflammatory damage in hepatocytes, and ultimately enables LBPs to exert remarkable anti-inflammatory and hepatoprotective effects.

### 4.5. Anti-Inflammatory Effects of LBPs on the Lungs

#### 4.5.1. Acute Lung Injury (ALI)

Acute lung injury (ALI) is a severe inflammatory condition caused by pulmonary or extrapulmonary insults, characterized by high mortality and clinically presenting as acute respiratory distress syndrome (ARDS), a destructive form of acute inflammatory lung disease [92]. In ALI, the activation and accumulation of inflammatory cells play a pivotal role. Therefore, anti-inflammatory effects are of significant importance in the treatment of ALI. Hong et al. [93] further confirmed the protective effects of LBP’s anti-inflammatory actions against hyperoxia-induced ALI. The study utilized C57BL/6 mice, which were exposed to 100% oxygen for 72 h to induce the ALI model. LBPs were administered via oral gavage at a dose of 100 mg/kg. The study demonstrated that LBPs markedly upregulate the protein expression of SIRT1, which further suppresses the oligomerization of NLRP3. LBPs subsequently block the recruitment of adaptor protein ASC and the activation of caspase-1, thereby restraining the excessive release of pro-inflammatory cytokines and ameliorating inflammatory responses as well as pulmonary tissue injury in ALI.

Chen et al. [94] demonstrated that the anti-inflammatory properties of LBPs confer protection against lipopolysaccharide (LPS)-induced ARDS. In this study, C57BL/6 mice were used, and the ARDS model was established by intratracheal instillation of 50 µL of sterile PBS solution containing 5 mg/kg LPS. LBPs were administered via oral gavage at a dose of 200 mg/kg. The study demonstrated that LBPs protect against ARDS-associated lung injury by reducing TNF-*α* and IL-6 levels in lung tissue and inhibiting LPS-induced phosphorylation of I *κ* B*α* and NF-*κ* B p65. This inhibition suppresses NF-*κ*B activation and reduces the release of inflammatory cytokines, thereby alleviating the inflammatory response in ARDS.

#### 4.5.2. Viral Pneumonia

Influenza A virus (IAV) is highly pathogenic to humans and has caused multiple pandemics, characterized by severe complications such as excessive inflammation and tissue damage during the infection process [95]. Therefore, mitigating the indirect damage caused by excessive inflammation is an effective therapeutic strategy. *L. barbarum* glycopeptide (LbGp) is a mixture of five highly branched polysaccharide-protein conjuncts (LbGp1-5) isolated from *L. barbarum* fruit, which was found by Li et al. [96] to exert therapeutic effects on H1N1 viral pneumonia caused by influenza A virus infection, attributed to its anti-inflammatory properties. The study used ICR mice, infecting them with the H1N1 viral strain A/PR/8/34 via intranasal instillation of 35 µL to induce viral pneumonia. LbGp was administered via oral gavage at low and high doses of 25 mg/kg and 50 mg/kg, respectively. This study confirmed that LbGp exerts anti-inflammatory effects via blockade of NF-*κ*B p65 nuclear translocation. By upregulating cytoplasmic I*κ*B*α* expression and inhibiting I*κ*B*α* phosphorylation and degradation, LbGp enhances the binding of cytoplasmic I*κ*B*α* to NF-*κ*B p65 to hamper p65 nuclear trafficking, which reduces the generation of downstream proinflammatory cytokines IL-1*β*, IL-6 and TNF-*α*. Moreover, LbGp restrains M1 macrophage polarization by lowering NOS2 expression and boosts M2 macrophage differentiation by increasing Arg-1 abundance, relieving excessive inflammation in H1N1-challenged mice.

#### 4.5.3. Allergic Asthma and Pneumonia

In the study investigating the effects of LBPs on lung injury and inflammation in mice with OVA-induced allergic asthma, Ren et al. [97] induced an allergic asthma model in C57BL/6 mice by ultrasonic nebulization inhalation of 50 µL of sterile saline solution containing 100 µg of OVA. LBPs were administered via oral gavage at a dose of 100 mg/kg. Cui et al. [98] induced an allergic asthma model in C57BL/6 mice by intranasal instillation of 50 µL of a 1 mg/mL OVA solution. LBPs were administered via oral gavage at a dose of 100 µg/g. Both studies demonstrated that LBPs elevate the relative abundance of beneficial intestinal bacteria including Lactobacillus and Bifidobacterium, while decreasing the proportion of pathogenic microbes. Such gut microbiota remodeling suppresses the secretion of pro-inflammatory cytokines TNF-*α*, IL-1*β* and IL-6, alleviates inflammatory cell infiltration and excessive mucus secretion in airway tissues, and ultimately ameliorates pulmonary lesions and inflammatory disorders in allergic asthmatic mice.

Furthermore, Bai et al. [99] demonstrated that LbGp exerts protective effects against allergic asthma induced by Artemisia pollen. The study employed BALB/c mice. Sensitization was achieved by intraperitoneal injection of 0.1 mL of Artemisia pollen sensitizing solution. Seven days later, the allergic asthma response was elicited by intranasal instillation of 20 µL of Artemisia pollen extract. LbGp was administered via oral gavage at three doses: low (2 mg/kg), medium (5 mg/kg), and high (10 mg/kg). The study demonstrated that a high dose of LbGp (10 mg/kg) exhibited the most significant therapeutic effects on allergic asthma. LbGp not only modulated the gut microbiota to regulate the levels of inflammation-associated cytokines but also reduced the infiltration of inflammatory cells (*p* < 0.0001) and decreased the proportion of Th17 cells, which play a pro-inflammatory role in allergic responses. Consequently, these actions alleviated the inflammatory response and mitigated lung tissue damage associated with allergic asthma.

### 4.6. Anti-Inflammatory Effects of LBPs on the Colon

#### 4.6.1. DSS-Induced Ulcerative Colitis

Li et al. [100] investigated the effects of LBPs on alleviating chronic ulcerative colitis (UC) induced by dextran sulfate sodium (DSS). Male C57BL/6 mice were subjected to a 2.0% (*w*/*v*) DSS solution for one week, followed by regular water for 14 days; this cycle was repeated three times to establish a chronic UC model [101]. LBPs were administered via rodent chow supplemented with 1% LBPs throughout the entire period of chronic colitis induction. Studies have shown that LBPs supplementation can reverse the significant increase in the expression of pro-inflammatory factors IL-1*β*, IL-6 and TNF-*α* in the distal colonic tissues of UC mice (*p* < 0.05), and elevate the expression of the anti-inflammatory cytokine IL-10 (*p* < 0.05), thereby alleviating the inflammatory response in DSS-induced chronic UC. Zhang et al. [102] established a UC model by providing C57BL/6 mice with drinking water containing 2.5% (*w*/*v*) DSS for 6 days. LBPs were administered via oral gavage at a dose of 0.8 g/kg body weight for 28 days. Further studies indicated that LBPs modulate the composition of the gut microbiota by reducing the abundance of pro-inflammatory bacteria associated with inflammation and increasing the abundance of beneficial bacteria, thereby regulating the levels of inflammation-associated cytokines and alleviating UC symptoms.

Studies have shown that during inflammatory bowel disease (IBD), M1 macrophages in the gut secrete pro-inflammatory cytokines such as IL-6, TNF-*α*, and IL-1*β*, contributing to inflammation. Conversely, M2-like macrophages secrete anti-inflammatory cytokines, including IL-10, and inhibit the secretion of pro-inflammatory cytokines, thereby mitigating the inflammatory response in IBD and facilitating its resolution [103]. Wang et al. [104] induced an IBD mouse model by administering 2.5% DSS to male C57BL/6 mice for 7 days. The mice were then treated with LBPs via gavage at doses of 200 mg/kg and 100 mg/kg, respectively. The study further confirmed that LBPs ameliorate IBD inflammation by modulating macrophage polarization. Specifically, in colon tissues from colitis mice, LBPs lower p-STAT1 to restrain STAT1 nuclear translocation and transcriptional activity, thereby suppressing M1 polarization, reducing NOS2 expression and M1 macrophage counts. On the other hand, LBPs elevate p-STAT6; the accumulated activated p-STAT6 undergoes nuclear translocation to facilitate M2 macrophage differentiation, upregulate Arg-1 expression, and expand the M2 macrophage population. LBPs remodel macrophage polarization homeostasis via reciprocal regulation of STAT1/STAT6 pathways, tune downstream inflammatory cytokine secretion, and significantly alleviate colitis-related pathological lesions, with the high-dose group (200 mg/kg) showing the most pronounced effects.

Capsaicin (CAP), a member of the vanilloid family, induces analgesic effects through its binding to the transient receptor potential vanilloid type 1 (TRPV1) channel [105]. Chen et al. [106] demonstrated that the combination of LBPs and CAP produces a synergistic effect in mitigating the inflammatory response and relieving pain in UC. The study induced a UC model in rats by administering 5% DSS in drinking water for 6 days. Subsequently, the rats were treated with 50 mg/kg LBPs and 12 mg/kg CAP via gavage. Collective experimental data demonstrate that combined treatment with LBPs and CAP exerts dual protective effects on colonic tissues. On one hand, the co-intervention blocks the nuclear translocation of NF-*κ*B and downregulates the transcriptional levels of TNF-*α* and IL-6 in colon tissues, thus mitigating colonic inflammatory responses. On the other hand, CAP + LBPs relieve pain sensation via suppressing the protein expression of TRPV1 and TRPA1, which ultimately ameliorates colonic pathological lesions.

Moreover, specific subtypes of LBPs, namely the homogeneous arabinogalactan LBP-3 and LBP-m isolated from goji berries, were shown to mitigate intestinal injury and inflammatory responses induced by DSS in UC, as demonstrated in studies by Cao et al. [107] and Mou et al. [108]. In the study by Cao C et al., a UC model was induced in C57BL/6J mice by administering 1.5% DSS in drinking water. The mice were then treated with 50 mg/kg LBP-3 via gavage. The study demonstrated that LBP-3 ameliorated DSS-induced colitis by downregulating the expression of TLR4 and MyD88, and by inhibiting the dephosphorylation of NF-*κ*B. In the study by Mou et al., a UC model was induced in C57BL/6J mice by administering 3% DSS in drinking water. The mice were treated with 100 mg/kg LBP-m via gavage. The study demonstrated that LBP-m mitigated the inflammatory response by downregulating the gene expression of TLR4, MyD88, and NF-*κ*B p65, thereby inhibiting the NF-*κ*B signaling pathway and reducing the production of inflammatory cytokines.

#### 4.6.2. Inflammatory Response of Colorectal Cancer Cells

Li et al. [109] examined the effects of LBPs on improving intestinal barrier dysfunction and inflammatory responses in human colorectal cancer (Caco-2) cells. Caco-2 cells were cultured in DMEM medium supplemented with 50 U/mL penicillin, 50 U/mL streptomycin, 10% fetal bovine serum (FBS), and 1% non-essential amino acids under conditions of 5% CO_2_, 95% relative humidity, and 37 °C [110]. The Caco-2 cells were seeded into 96-well cell culture plates at a density of 5 × 10^3^ cells per well and incubated for 24 and 48 h under the same conditions. TNF-*α* (100 ng/mL) was added to the basolateral side, followed by the addition of different concentrations of LBPs to the wells. After 24 h of incubation, paracellular permeability, TER values, and pro-inflammatory markers were measured. Experimental results reveal that LBPs at a concentration of 400 µg/mL exert prominent protective effects against TNF-*α*-induced intestinal barrier dysfunction. Mechanistically, LBPs block the upstream initiating signal mediated by TNF-*α* and further hinder midstream activation of the NF-*κ*B signaling cascade. Such sequential suppression ultimately downregulates the secretion of NF-*κ*B-dependent downstream pro-inflammatory cytokines, thereby ameliorating intestinal barrier impairment and alleviating intestinal inflammatory responses.

**Figure 6:**
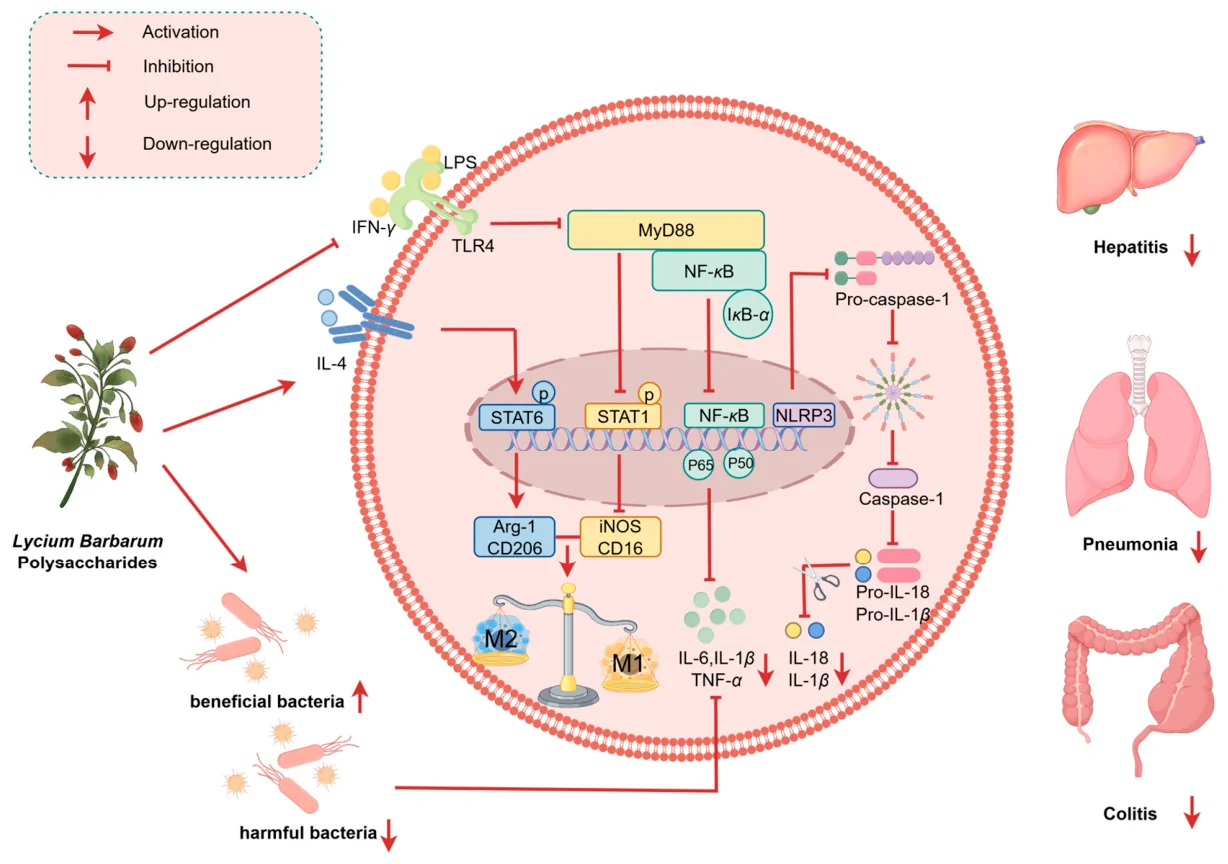
The main anti-inflammatory mechanism of LBPs on liver, lung and colon.

LBPs exert robust anti-inflammatory and tissue-protective functions in the liver, lungs, and colon via the comprehensive regulation of inflammatory gene transcription, core signaling cascades, and macrophage phenotypic polarization. Mechanistically, LBPs downregulate mRNA levels of upstream SIRT1, SIRT6 and NF-*κ*B p65 to block inflammatory signaling initiation, thus reducing downstream secretion of proinflammatory cytokines including IL-1*β*, IL-4, IL-6, TNF-*α* and IFN-*γ*. For NLRP3 inflammasome-mediated pyroptosis, LBPs sequentially decrease NLRP3, caspase-1 and GSDMD-N protein expression to restrain inflammasome overactivation and pyroptosis. LBPs also inhibit excessive NF-*κ*B activation to relieve persistent inflammation in hepatic, pulmonary and colonic tissues. In terms of macrophages, LBPs lower p-STAT1 to decrease M1 macrophages and NOS2 expression, while elevating p-STAT6 to increase M2 macrophages and Arg-1 abundance. Collectively, this hierarchical upstream-to-downstream regulation balances proinflammatory and anti-inflammatory immunity and alleviates inflammatory damage in three organs. The comprehensive anti-inflammatory mechanism of LBPs is presented in Figure 6.

### 4.7. Anti-Inflammatory Effects of LBPs on Other Organs

Xiong et al. [111] examined the protective effects of LBPs in a mouse model of cerulein-induced acute pancreatitis. Acute pancreatitis was induced in C57BL/6 mice by intraperitoneal injection of 50 mg/kg cerulein. The mice were subsequently treated with 100 mg/kg LBPs via gavage. The study demonstrated that LBPs mitigated pancreatic inflammation by reducing inflammatory cell infiltration and the release of inflammatory cytokines and proteases in pancreatic tissues. LBPs also decreased serum levels of TNF-*α* and IL-6, thereby attenuating the inflammatory response, reducing pancreatic tissue damage, and alleviating the symptoms of acute pancreatitis.

Liu et al. [112] investigated the protective effects of LBPs against LPS-induced acute peritonitis in male BALB/c mice. Acute peritonitis was induced by intraperitoneal injection of 20 mg/kg LPS. The mice were then treated with LBPs via gavage at low (400 mg/kg) and high (800 mg/kg) doses. The study demonstrated that the high dose of LBPs (800 mg/kg) had a more significant anti-inflammatory effect. LBPs promoted the shift in macrophages from the pro-inflammatory M1 phenotype to the anti-inflammatory M2 phenotype, thereby inhibiting macrophage pro-inflammatory activity and reducing the inflammatory response. Moreover, LBPs downregulated the expression of TLR4, NF-*κ*B p65, and I*κ*B*α* induced by LPS, which in turn decreased serum levels of pro-inflammatory cytokines TNF-*α*, IL-1*β*, and IL-6. These actions collectively reduced the production of inflammatory mediators and provided protection against acute peritonitis in mice.

**Table 3:** Anti-inflammatory effects and mechanisms of LBPs on inflammation in different organs.

Organs	Animal Modeling	In Vitro/In Vivo	LBPs Concentration	Concomitant Drugs/Derivatives	Action or Mechanism	Ref
Ocular	Corneal injury induced by NaOH alkali burn in SD rats	In vivo	2 mg/mL		Attenuate the mRNA expression levels of TNF-*α*, CASP3, and MMP9, and inhibit the activation of the IL-17 signaling pathway	[73,74,75,76,77]
	DED-induced by combined DS and BAC in C57BL/6 mice	In vivo	12.5 mg/mL			
	UVB-induced HaCa T Cell injury	In vitro	300 µg/mL		Inhibit the phosphorylation of p38 MAPK, attenuate the activation of caspase-3, and downregulate the expression of MMP-9	
	IR-induced corneal injury in C57BL/6 mice	In vivo		PLBP 10 mg/mL	Reduce the protein levels of TNF-*α*, IL-1*β*, p-I *κ* B-*α*, and p-NF-*κ*B-p65, and inhibit the activation of the NF-*κ*B signaling pathway	
Kidney	HFD/STZ-induced DN in C57BL/6 mice	In vivo	80 mg/kg		Inhibit the translocation of NF-*κ*B p65 from the cytoplasm to the nucleus, and suppress the pro-inflammatory cascade	[78,79,80,113]
	LPS-induced sepsis in SD rats	In vivo	800 mg/kg		Inhibit the production and release of TNF-*α* and IL-1*β*, and reduce the protein expression levels of NF-*κ*B and Keap1	
	Lead-induced renal injury in ICR mice	In vivo	400 mg/kg			
Skeletal joint	Type II collagen-induced CIA in DBA/1 mice	In vivo	100 mg/kg		Reduce the levels of TNF-*α* and IL-17, restore the levels of IL-10, increase the relative abundance of beneficial bacteria, and decrease the relative abundance of harmful bacteria	[81,82,83,84]
	Type II collagen-induced RA in Wistar rats	In vivo	400 mg/kg			
Liver	Diet-deficient-induced NASH in C57BL/6N mice	In vivo	1 mg/kg		Inhibit the expression of NF-*κ*B-p50, NLRP3, TLR4 and p38 MAPK, reduce the levels of IL-1*β* and IL-18, and suppress the activation of the NF-*κ*B, NLRP3/6 inflammasome, TLR4 and MAPK signaling pathways	[85,86,87,88,89,90,91]
	High-fat diet-induced NASH in SD rats	In vivo	100 mg/kg	AE 20 m/min 60 min/d		
		In vivo	50 mg/kg	AE 33 cm/s 60 min/d		
	CCl_4_-induced hepatitis in C57BL/6N mice	In vivo	1 mg/kg		Reduce the mRNA expression of TNF-*α*, IL-1*β*, NLRP-3 and MIP-1, and inhibit the activation of the NLRP-3 inflammasome	
	EtOH-induced injury in L-02 Cells	In vitro	48 μg/mL			
Pulmonary	Hyperoxia-induced ALI in C57BL/6 mice	In vivo	100 mg/kg		Inhibit the levels of TNF-*α*, IL-6 and IL-1*β*, and the phosphorylation of I*κ*B*α* and NF-*κ*B p65, and inhibit the activation of the NLRP3 inflammasome and NF-*κ*B	[92,93,94,95,96,97,98,99]
	LPS-induced ARDS in C57 BL/6 mice	In vivo	200 mg/kg			
	Viral pneumonia in ICR mice infected with H1N1 Virus Strain A/PR/8/34	In vivo		LbGp 50 mg/kg	Inhibit the polarization of pro-inflammatory M1 macrophages, and induce the polarization of anti-inflammatory M2 macrophages	
	OVA-induced allergic asthma in C57BL/6 mice	In vivo	100 mg/kg		Increase the proportion of beneficial bacteria in the gut, decrease the proportion of harmful bacteria, and reduce the proportion of pro-inflammatory Th17 cells	
		In vivo	100 μg/g			
	Allergic asthma in BALB/c mice induced by artemisia pollen	In vivo		LbGp 10 mg/kg		
Colon	DSS-induced UC in C57BL/6 mice	In vivo	1%		Decrease the abundance of pro-inflammatory bacteria, and increase the abundance of beneficial bacteria to regulate the levels of inflammatory cytokines; reduce the levels of p-STAT1, M1 macrophages and NOS2, and increase the levels of p-STAT6, M2 macrophages and Arg-1; decrease the gene expression levels of TLR4 and NF-*κ*B p65, and inhibit the NF-*κ*B signaling pathway	[100,101,102,103,104,105,106,107,108,109,110]
		In vivo	0.8 g/Kg			
		In vivo	200 mg/kg			
	DSS-induced UC in SD mice	In vivo	50 mg/kg	CAP 12 mg/kg		
	DSS-induced UC in C57BL/6 mice	In vivo		LBP−3 50 mg/kg		
		In vivo		LBP-m 100 mg/kg		
	TNF-*α*-induced inflammation in Caco-2 cells	In vitro	400 μg/mL		Alleviate the upregulation of MLCK protein expression and MLC phosphorylation, and inhibit the activation of NF-*κ*B	
Miscellaneous	Cerulein-induced acute pancreatitis in C57BL/6 mice	In vivo	100 mg/kg		Reduce inflammatory cell infiltration and decrease the levels of TNF-*α* and IL-6	[111]
	LPS-induced acute peritonitis in BALB/c mice	In vivo	800 mg/kg		Promote the polarization of macrophages from pro-inflammatory M1 to anti-inflammatory M2, and inhibit the expression of TLR4	[112]

## 5. Toxicity of LBPs

Drug toxicity testing serves as the “first line of defense” to safeguard human medication safety. It is a scientific research method widely applied in fields such as new drug development and chemical safety evaluation. As a traditional Chinese medicinal material with homologous medicinal and edible properties, the toxicity of LBPs—a natural product derived from *L. barbarum* fruits—has attracted considerable attention. Toxicity experiments play a crucial role in the research and application of new drugs; meanwhile, they provide essential references for the design of clinical medication doses or the formulation of exposure limits.

### 5.1. Acute Toxicity Experiments

Yan et al. [114] investigated the acute toxicity of LBPs in aged mice. To induce an aged mouse model, the mice were intraperitoneally injected with 50 mg/kg D-galactose daily for 6 weeks. For the acute toxicity study, the mice were administered LBPs via gavage at the maximum concentration and volume in a single dose once daily for 7 days. Throughout the study period, the toxicity reactions and side effects in the mice were observed and recorded. The experimental results indicated that the mice exhibited smooth fur, normal fecal color and texture, and normal activity. No deaths or other abnormalities were observed during the one-week period of gavage administration. Furthermore, no significant changes or abnormalities were noted in the appearance of the heart, lungs, spleen, liver, and kidneys. These findings suggest that LBPs are non-toxic to aged model mice, indicating that goji berry polysaccharides are non-toxic substances.

Zhao et al. [115] conducted an acute toxicity study of LBP capsules in mice. A single maximum dose was administered orally at 20 g/kg, with a volume of 0.4 mL/10 g, to 20 healthy Kunming mice (equally divided between males and females). The mice were observed continuously for 7 days. Following administration, no signs of distress or other abnormal reactions were observed, no deaths occurred, and the mice maintained normal activity. There was no increase in secretions, and the fur remained clean. Upon dissection, the weight, texture, and color of the organs were found to be normal. These results suggest that LBP capsules are essentially safe and non-toxic for both male and female mice.

Zhao et al. [116] investigated the acute toxicity of LBPs. The median lethal dose (LD50) was determined using the Karber’s method, with a dose range of 40 g/kg to 7 g/kg. Mice were intraperitoneally injected with 0.4 mL of LBPs solution, and their mortality, clinical manifestations, and pathological changes were observed over a 7-day period. The results indicated that the acute toxicity LD50 of LBPs for mice was 20.42 g/kg. Since this value exceeds 1.5 g/kg, LBPs are classified as a non-toxic substance.

Wu et al. [117] investigated the acute toxicity of LBPs to goldfish. Ten goldfish were randomly placed into an aquarium containing 10 L of LBPs solution. Following the static water bioassay method, the solution was not changed during the test period, continuous aeration was maintained, and no food was provided. The behavior, toxic symptoms, and mortality of the goldfish were observed throughout the experiment. The results indicated that the 24, 48, 72, and 96 h LC50 values were 2173.84, 1705.61, 1594.19, and 1568.08 mg/L, respectively. The safe concentration was determined to be 278.92 mg/L. These findings suggest that LBPs are safe for goldfish within the safe concentration range.

### 5.2. Long-Term Toxicity Experiments

Zhao et al. [115] conducted a long-term toxicity study of LBP capsules in mice. The mice were administered LBP capsules via gavage at three doses: low (2.5 g/kg), medium (5 g/kg), and high (10 g/kg). Administration was performed daily for 24 weeks. The study observed the mice’s physical signs, fecal characteristics, and behavior, as well as changes in body weight, biochemical blood parameters, and histopathological changes. The results indicated that no deaths occurred among the mice, and their activity, feeding, and excretion remained normal. Histopathological examination of major organs, including the heart, liver, spleen, lungs, and kidneys, revealed no significant abnormalities. These findings suggest that long-term administration of LBP capsules is non-toxic to mice.

Taken together, systematic toxicological assessments including acute and long-term toxicity tests have verified the high biosafety of LBPs. Within the experimental dose range, no adverse toxic reactions or organ damage were observed in experimental animals, and no histopathological lesions were detected in major organs after long-term administration. These data demonstrate that LBPs have extremely low toxicity and favorable safety performance, which provides sufficient scientific evidence for its further development and application in functional foods and pharmaceutical fields. Relevant toxicity data of LBPs are presented in Table 4.

## 6. Application of LBPs

**Figure 7:**
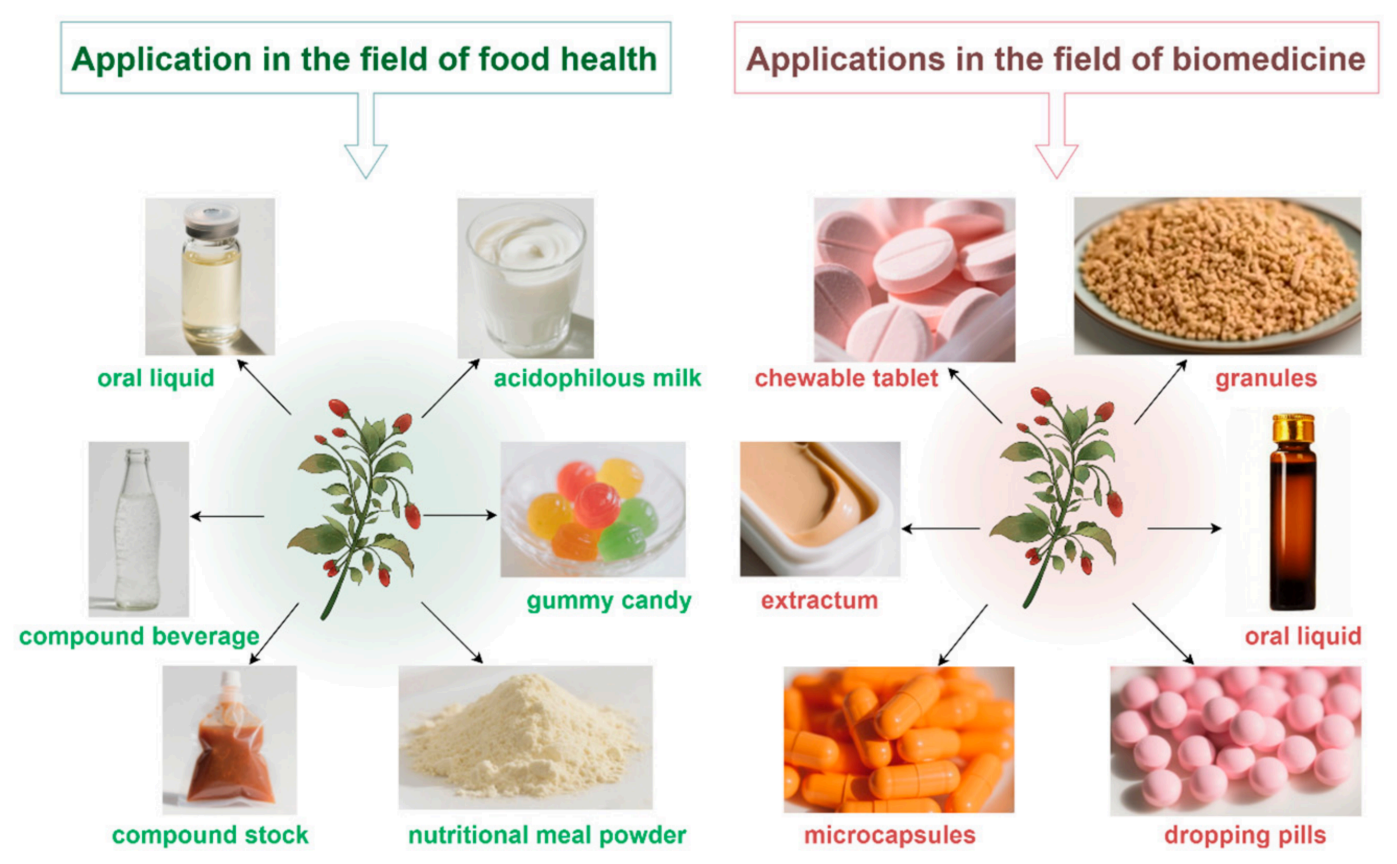
Applications of LBPs in the food and healthcare field and biomedical field.

As a naturally occurring bioactive polysaccharide with favorable safety profiles, LBPs exert prominent anti-inflammatory effects across multiple organs. The underlying mechanisms include regulation of inflammatory genes, blockade of core signaling pathways, suppression of inflammasome activation, and modulation of macrophage polarization. Accordingly, LBPs have attracted extensive research interest in functional food and biomedical sciences. The applications of LBPs in food healthcare and biomedicine are presented in Figure 7.

### 6.1. Food and Healthcare Field

LBPs show broad application potential in the food and healthcare field due to their significant anti-inflammatory effect. For example, LBPs combined oral liquid is formulated by compounding LBPs with anthocyanins, catechins, lutein, and other components. It can protect the eyes from infections and alleviate ocular inflammation, making it suitable for people who use electronic screens for a long time [118]. By combining LBPs with milk for fermentation, a functional yogurt with liver-protecting and hangover-relieving effects is prepared. This yogurt can not only improve inflammatory conditions in the liver but also preventively reduce liver damage and fatty liver. Additionally, it has an excellent flavor and texture, making it suitable for people of all ages [119]. There is also a composite polysaccharide beverage made by combining LBPs with Chinese yam polysaccharides and chrysanthemum polysaccharides. It can reduce the increase in intestinal inflammatory factors caused by intestinal damage and enhance the intestinal barrier function [120]. In addition, *L. barbarum* compound original pulp adopts the molecular-level compatibility of LBPs, cordycepin from Cordyceps militaris, and emblica polyphenols to achieve metabolic complementarity and functional synergy among the three components. At the same time, it improves the bioavailability of LBPs and enhances the body’s anti-inflammatory effect [121]. Furthermore, there are LBPs gel candies, which have a soft and chewy texture. They can improve the intestinal microbiota and are healthy leisure foods with healthcare functions [122]. The nutritional meal powder prepared from three dual-purpose (food and medicine) herbal powders—LBPs, longan polysaccharides, and jujube polysaccharides—can improve intestinal function, regulate the intestinal microbiota, and promote body health [123].

### 6.2. Biomedical Field

As a natural product derived from a dual-purpose (food and medicine) material, LBPs have been widely used in the biomedical field due to its mild medicinal properties and safety (non-toxicity). For example, in eye care applications, LBPs chewable tablets, prepared by combining LBPs with blueberry concentrate powder and Aronia melanocarpa fruit, can improve blurred vision and dry eyes, as well as relieve eye fatigue [124]; granules made from the combination of LBPs and chrysanthemum extract can reduce inflammatory responses in ocular tissues and protect visual function [125]; additionally, wolfberry paste containing LBPs is used as a retinal neurotrophic agent to prevent and treat ocular diseases [126]. In addition, LBPs microcapsules are prepared by using the compound polysaccharides of LBPs and konjac glucomannan as the wall material to encapsulate octacosanol and pueraria flavonoids. They can improve alcoholic liver injury and alleviate alcohol-induced inflammatory responses in the liver [127]. Furthermore, there are LBPs dropping pills that can inhibit pathogens causing respiratory infections and regulate inflammatory responses in the lungs, which are used for the prevention and treatment of coronavirus infections [128]. The oral liquid of LBPs and betaine self-assembled nanoparticles exhibits excellent anti-inflammatory activity in vitro and in vivo, promotes the treatment of colitis, and can also achieve the effects of enhancing efficacy and reducing toxicity [129].

In addition, LBPs exert an improving effect on cognitive impairment and systemic inflammatory levels in mice with neuroinflammation induced by a high-fat, high-fructose diet (HFFD), and can be used in the preparation of various pharmaceutical formulations [130]. LBPs can also be applied in drugs designed to alleviate microglial inflammation or neuroinflammation [131]. Furthermore, inventions and research have revealed that LBPs can be used in drugs that inhibit the secretion of inflammatory factors, as it can reduce the levels of TNF-*α*, IL-1*β*, and IL-6 [132].

## 7. Summary and Outlook

This review comprehensively elaborates on the green extraction optimization, structural characteristics, multi-organ anti-inflammatory mechanisms, toxicological safety, and application potential of LBPs. Benefiting from the continuous advancement of modern auxiliary extraction technologies and statistical optimization strategies, including response surface methodology, ultrasonic–microwave synergistic extraction, ultrasonic-assisted enzymatic extraction, and green solvent substitution, the extraction yield, structural integrity, purification efficiency, and environmental sustainability of LBPs have been greatly improved, providing high-quality raw material support for subsequent mechanistic exploration and product development. The unique molecular weight distribution, specific monosaccharide composition, and glycosidic linkage patterns endow LBPs with stable and superior biological activities. As a typical multi-target natural anti-inflammatory agent, LBPs exert protective effects against inflammatory damage in multiple organs, covering the eyes, kidneys, bone joints, liver, lungs, and colon. Mechanistically, LBPs alleviate local and systemic inflammatory responses by suppressing the transcription of inflammation and apoptosis-related genes, reducing the secretion of pivotal pro-inflammatory cytokines, inhibiting the overactivation of NF-*κ*B, MAPK, and NLRP3 inflammasome signaling cascades, balancing M1/M2 macrophage phenotypic polarization, and restoring intestinal microecological homeostasis. Both acute and long-term toxicological studies have verified that LBPs possess extremely low toxicity and excellent biosafety, with no adverse physiological responses or histopathological lesions observed in vivo. Consistent with existing research trends, bibliometric analysis further confirms that anti-inflammatory regulation and translational application exploration remain the core research hotspots and developmental directions of the LBPs field.

In terms of the clinical intervention of inflammatory diseases, conventional synthetic anti-inflammatory drugs, such as non-steroidal anti-inflammatory drugs (NSAIDs) and glucocorticoids, can rapidly alleviate acute inflammatory symptoms. However, long-term or high-dose administration frequently causes inevitable side effects, including gastrointestinal irritation, hepatic and renal toxicity, immune dysfunction, and drug resistance, which greatly limit their long-term application in chronic inflammatory disorders. In comparison, LBPs exhibit prominent advantages as a natural alternative and adjuvant anti-inflammatory agent. Distinct from the single-target working pattern of chemical drugs, LBPs modulate inflammatory progression through multi-pathway and multi-dimensional regulatory networks, achieving systematic homeostasis of inflammatory signaling, immune function, and intestinal microecology. Notably, LBPs feature favorable biocompatibility, negligible toxic side effects, and cumulative protective efficacy, rendering it suitable for the long-term intervention of chronic low-grade inflammation, immune-mediated inflammation, and multi-organ inflammatory injuries. Accordingly, LBPs effectively compensate for the clinical deficiencies of traditional anti-inflammatory agents and present unique application prospects in the prevention, auxiliary treatment, and rehabilitation of various chronic inflammatory diseases.

Nevertheless, current research still has several limitations that hinder the in-depth development and clinical transformation of LBPs. The precise structure–activity relationship of homogeneous LBPs fractions remains poorly clarified, and the upstream molecular targets as well as the crosstalk among multiple inflammatory signaling pathways require further systematic exploration. In addition, most existing studies are confined to in vitro cell experiments and in vivo animal models, while high-quality, large-sample clinical trials are still lacking. Relevant clinical evidence regarding the optimal dosage, long-term efficacy, and safety of LBPs in human inflammatory diseases is insufficient to support its clinical popularization. Furthermore, standardized industrial systems for LBPs purification, structural modification, and large-scale production have not been fully established, restricting its industrial transformation and commercial application.

Future studies should focus on the isolation and purification of homogeneous LBPs components to elucidate their precise structure–activity relationships and core anti-inflammatory molecular mechanisms. In-depth investigation of the interactive relationships among LBPs, inflammatory signaling networks, and intestinal flora will help further uncover its systematic regulatory mechanisms. Moreover, standardized clinical trials are urgently needed to validate the clinical translational value of LBPs for the intervention of chronic inflammatory diseases. Additionally, the establishment of green, efficient, and standardized industrial preparation techniques will greatly promote the comprehensive application of LBPs in functional food development, healthcare products, and biomedical adjuvant therapy, facilitating the transformation of LBPs from fundamental experimental research to clinical practice and large-scale industrialization.

## Figures and Tables

**Table 1:** Major structural parameters of purified LBPs and LRPs.

Name	Origin	Molecular Weights/Da	Monosaccharide Composition and Quantity Ratio of Substance	Structures (Backbone)	Analytical Techniques	Ref
LBP-1	*L. barbarum* L.	3.078 × 10^5^	Ara:Gal:Glc:Xyl:Man = 37.53:28.08:14.72:7.83:4.50	*α*-L-Ara(1→[5-*α*-L-Ara(1→3)-*β*-D-Galp-(1→]_n_→4)-*α*-D-Galp-(1[→5-*α*-L-Ara(1]_n_[→6)-*β*-D-Galp-(1→4)-*β*-D-Galp-(1→]_n_	methylation analysis, NMR	[25]
LBP-3	*L. barbarum*	6.74 × 10^4^	Ara:Gal = 1.00:1.56	1, 3-*β*-Galp, substituted at C-6	methylation analysis, NMR, FT-IR	[26]
LBP-W	*L. barbarum* L.	1.1297 × 10^5^	Ara:Gal:Rha = 55.6:35.5:8.0	→6)-*β*-D-Galp-(1→residues, repeatedly	methylation analysis, NMR, FT-IR	[27]
LBP3b	*L. barbarum* L.	4.92 × 10^3^	Rha:Xyl:Gal:Glc:Man = 5.11:1.70:1:28.06:5.52	Furan and pyran rings with *α* and *β* glycosidic bonds	NMR, FT-IR	[28]
LBP-S-1	*L. barbarum* L.	1.92 × 10^6^	Rha:Ara:Xyl:Man:Glc:Gal:GalA = 1.00:8.34:1.25:1.26:1.91:7.05:15.28	Furan and pyran rings with *α* and *β* glycosidic bonds	NMR, FT-IR	[29]
LBPA	*L. barbarum* L.	4.7 × 10^5^	Ara:Gal:GlcA:Rha = 9.2:6.6:1.0:0.9	(1→6)-*β*-D-Galactan	methylation analysis, NMR	[30]
LBP1A1-1	*L. barbarum* L.	4.5 × 10^4^	Ara:Gal:Glu:Rha = 47.8:49.8:1.4:1.2	1, 3-linked *β*-Galp, 1, 6-linked *β*-Galp, 1, 4-linked *α*-Glcp	methylation analysis, NMR, FT-IR	[31]
LBP1B-S-2	*L. barbarum* L.	8.00 × 10^4^	Rha:Ara:Gal:GlcA = 3.13:53.55:39.37:3.95	1,3-*β*-D-Galp, 1,6-*β*-D-Galp	methylation analysis, IR, NMR	[32]
LBP1C-2	*L. barbarum* L.	9.98 × 10^4^	Ara:Gal:Rha:GalA = 49.9:33.6:8.0:8.5	alternate 1, 2-linked *α*-Rhap and 1, 4-linked *α*-GalpA	methylation analysis, NMR	[33]
p-LBP	*L. barbarum* L.	6.40 × 10^4^	Fuc:Rha:Ara:Gal:Glc:Xyl:GalA:GlcA = 1.00:6.44:54.84:22.98:4.05:2.95:136.98:3.35	→4-*α*-GalpA-(1→, repeatedly	methylation analysis, NMR, FT-IR	[34]
LbGp1	*L. barbarum* L.	4.91 × 10^4^	Ara:Gal = 5.6:1	→6)Galp(1→linked galactose substituted at O-3 by galactosyl or arabinosyl groups	methylation analysis, ESI-MS	[35]
LBGP70	*L. barbarum* L.	9.5 × 10^4^	Ara:Gal:Rha:Glc = 63.7:26.8:4.1:2.4	(1→6)-*β*-Gal, substituted at the C-3 position	methylation analysis, NMR, FT-IR	[36]
XLBP-I-I	*L. barbarum* L.	4.196 × 10^5^	Ara:Rha:Xyl:Gal:GlcA:GalA = 26.5:12.9:0.7:16.8:2.3:40.8	alternate 1,2-*α*-L-Rhap and 1,4-*α*-D-GalpA	methylation analysis, NMR, FT-IR	[37]
LBLP5-A	*L. barbarum*	1.133 × 10^5^	Rha:Ara:Gal = 0.5:1.9:1.0	(1→3)-*β*-D-Galp, repeatedly	methylation analysis, FT-IR	[38]
LRGP1	*L. ruthenicum* Murr.	5.62 × 10^4^	Rha:Ara:Xyl:Man:Glu:Gal = 0.65:10.71:0.33:0.67:1:10.41	(1→3)-Gal	methylation analysis, ESI-MS	[39]
LRGP3	*L. ruthenicum* Murr.	7.56 × 10^4^	Rha:Ara:Gal = 1.0:14.9:10.4	*β*-D-(1→3)-Gal, substituted at the O-6 position by galactosyl or arabinosyl groups	methylation analysis, NMR, ESI-MS	[40]
LRGP5	*L. ruthenicum* Murr.	1.37 × 10^5^	Rha:Ara:Xyl:Gal:GalA = 1.0:2.2:0.5:0.5:1.2:4.7	(1→4)-linked galacturonic acid, interrupted by (1→2)-linked rhamnose	methylation analysis, NMR, ESI-MS	[41]
LRP3-S1	*L. ruthenicum* Murr.	1.148 × 10^5^	Rha:GalA:Gal:Xyl:Ara = 14.4:17.7:26.6:16.4:24.9	RG-I, substituted at C-4 of rhamnose units by side chains	methylation analysis, NMR	[42]
LRP-S2A	*L. ruthenicum* Murr.	2.65 × 10^6^	Rha:Ara:Gal:Glc:GlcA = 1.00:2.07:0.57:2.59:4.33	6-O-Me-*α*-(1→4)-D-GlcA, 2-O-acetyl-*α*-(1→4)-D-Glc, *α*-(1→2,4)-L-Rhap, *β*-(1→3)-D-Galp and *α*-(1→3,5)-L-Araf	methylation analysis, NMR, FT-IR	[43]
LRP4-A	*L. ruthenicum* Murr.	1.05 × 10^5^	Rha:Ara:Glu:Gal = 1:7.6:0.5:8.6	*β*-(1→6)-Galp	methylation analysis, IR, NMR, ESI-MS	[44]

**Table 2:** LBPs extraction process and advantages.

Methods	Polysaccharide Components	Temperature (°C)	Time (min)	Process Conditions	Total Yield (%)	Merits	Ref
Ultrasonic extraction method	LBPs	73	80	Solid–liquid ratio 1:38 g/mL	12.54 ± 0.12	High extraction efficiency Short extraction time	[54]
Ultrasound-enhanced subcritical water	LBPs	100	53	Solid–liquid ratio 1:26 g/mL Solvent subcritical water	5.728	Reduce environmental pollution and solvent residue Suitable for industrial production	[56]
Ultrasonic-microwave combined method	LLP		Microwave: 16 Ultrasonic: 20	Solid–liquid ratio 1:55 g/mL	1.873	High extraction efficiency Suitable for industrialized production	[59]
Ultrasonic-assisted enzymatic method	LBPs	55.79	20.29	Enzyme concentration 2.15%	6.31 ± 0.03	Mild and environmentally friendly Efficient and time-saving	[62]
Microwave-assisted compound enzymatic	LBPs	52	Enzyme: 150 Microwave: 4	Enzyme concentration 7%	24.37 ± 1.65	High extraction efficiency Short extraction time	[63]
Tissue crushing extraction-assisted aqueous two-phase system	LBPs	room temperature	4	Solid–liquid ratio 1:30 g/mL Solvent 17.86% (NH_4_)_2_SO_4_ and 28.86% EtOH	2.479	Efficient, green and economical Suitable for industrialized production	[64]
Temperature switchable deep eutectic solvent	LBPs	35	70	Solid–liquid ratio 1:25 g/mL Solvent DES-3 (tetracaine: lauric acid, 1:1)	46.5	High extraction efficiency Green, recyclable extraction	[69]

**Table 4:** Toxicity test of LBPs.

Experimental Type	Experimental Animal	Administration Concentration	Administration Route	Administration Duration	Experimental Result	Result Evaluation	Ref
Acute toxicity	Aged mice	-	Maximum concentration, maximum volume, single oral gavage administration	7 days	No obvious abnormal changes in mice	LBPs is non-toxic substance	[114]
Acute toxicity	Kunming mice	20 g/kg, 0.4 mL/10 g	Single maximum limit oral administration	7 days	No abnormalities observed in mice	LBP capsules are basically safe and non-toxic	[115]
Acute toxicity	Mice	40 g/kg~7 g/kg, 0.4 mL	Intraperitoneal injection administration (LD_50_ determined by Karber’s method)	7 days	No abnormalities observed in mice	LBPs are considered non-toxic.	[116]
Acute toxicity	Goldfish	10 L of LBPs solution	-	24, 48, 72, 96 h	LC_50_ values were 2173.84, 1705.61, 1594.19, and 1568.08 mg/L respectively	LBPs are safe for goldfish within the range of 278.92 mg/L	[117]
Chronic toxicity	Mice	2.5 g/kg, 5 g/kg, 10 g/kg	Oral gavage administration	24 weeks	No obvious abnormal changes in mice	Long-term administration of LBP capsules shows no obvious toxicity	[115]

## Data Availability

No new data were created or analyzed in this study. Data sharing is not applicable to this article.

## Abbreviations

The following abbreviations are used in this manuscript:

Ara	Arabinose
Araf	Arabino furanose
Fuc	Fucose
Gal	Galactose
GalA	Galacturonic acid
Galp	Galactopyranose
GalpA	Galactopyranuronic acid
Glc	Glucose
GlcA	Glucuronic acid
Glcp	Glucopyranose
Glu	Glucose
Man	Mannose
Rha	Rhamnose
Rhap	Rhamnopyranose
Xyl	Xylose
